# *Shank3* deletion in PV neurons is associated with abnormal behaviors and neuronal functions that are rescued by increasing GABAergic signaling

**DOI:** 10.1186/s13229-023-00557-2

**Published:** 2023-08-01

**Authors:** Jessica Pagano, Silvia Landi, Alessia Stefanoni, Gabriele Nardi, Marica Albanesi, Helen F. Bauer, Enrico Pracucci, Michael Schön, Gian Michele Ratto, Tobias M. Boeckers, Carlo Sala, Chiara Verpelli

**Affiliations:** 1 CNR, Neuroscience Institute, Via Follereau 3, 20854 Vedano al Lambro, Milan, Italy; 2 CNR, Neuroscience Institute, Pisa, Italy; 3 NEST, Istituto Nanoscienze-CNR and Scuola Normale Superiore, Pisa, Italy; 4 Institute for Anatomy and Cell Biology, Ulm University, Ulm, Germany; 5 Padova Neuroscience Center, Università degli Studi di Padova, Padua, Italy; 6 DZNE, Ulm Site, Ulm, Germany

**Keywords:** Autism, Hyperexcitability, Ganaxolone, GABA_A_ receptor

## Abstract

**Background:**

Phelan–McDermid syndrome (PMS) is a neurodevelopmental disorder characterized by developmental delay, intellectual disability, and autistic-like behaviors and is primarily caused by haploinsufficiency of SHANK3 gene. Currently, there is no specific treatment for PMS, highlighting the need for a better understanding of SHANK3 functions and the underlying pathophysiological mechanisms in the brain. We hypothesize that SHANK3 haploinsufficiency may lead to alterations in the inhibitory system, which could be linked to the excitatory/inhibitory imbalance observed in models of autism spectrum disorder (ASD). Investigation of these neuropathological features may shed light on the pathogenesis of PMS and potential therapeutic interventions.

**Methods:**

We recorded local field potentials and visual evoked responses in the visual cortex of *Shank3*∆11^−/−^ mice. Then, to understand the impact of *Shank3* in inhibitory neurons, we generated Pv-cre^+/−^ *Shank3*^Fl/Wt^ conditional mice, in which *Shank3* was deleted in parvalbumin-positive neurons. We characterized the phenotype of this murine model and we compared this phenotype before and after ganaxolone administration.

**Results:**

We found, in the primary visual cortex, an alteration of the gain control of *Shank3* KO compared with Wt mice, indicating a deficit of inhibition on pyramidal neurons. This alteration was rescued after the potentiation of GABA_A_ receptor activity by Midazolam. Behavioral analysis showed an impairment in grooming, memory, and motor coordination of Pv-cre^+/−^ *Shank3*^Fl/Wt^ compared with Pv-cre^+/−^ *Shank3*^Wt/Wt^ mice. These deficits were rescued with ganaxolone, a positive modulator of GABA_A_ receptors. Furthermore, we demonstrated that treatment with ganaxolone also ameliorated evocative memory deficits and repetitive behavior of *Shank3* KO mice.

**Limitations:**

Despite the significant findings of our study, some limitations remain. Firstly, the neurobiological mechanisms underlying the link between Shank3 deletion in PV neurons and behavioral alterations need further investigation. Additionally, the impact of Shank3 on other classes of inhibitory neurons requires further exploration. Finally, the pharmacological activity of ganaxolone needs further characterization to improve our understanding of its potential therapeutic effects.

**Conclusions:**

Our study provides evidence that Shank3 deletion leads to an alteration in inhibitory feedback on cortical pyramidal neurons, resulting in cortical hyperexcitability and ASD-like behavioral problems. Specifically, cell type-specific deletion of Shank3 in PV neurons was associated with these behavioral deficits. Our findings suggest that ganaxolone may be a potential pharmacological approach for treating PMS, as it was able to rescue the behavioral deficits in Shank3 KO mice. Overall, our study highlights the importance of investigating the role of inhibitory neurons and potential therapeutic interventions in neurodevelopmental disorders such as PMS.

**Supplementary Information:**

The online version contains supplementary material available at 10.1186/s13229-023-00557-2.

## Background

Haploinsufficiency of SHANK3 is widely recognized as the major cause of Phelan–McDermid syndrome (PMS), a complex neurodevelopmental disorder [1]. Major clinical features of the syndrome include global developmental delay, moderate-to-severe intellectual impairment, absent or delayed speech and neonatal hypotonia. Over 80% of patients with PMS exhibit autistic-like behavior including impaired communication and social interaction, poor eye contact, anxiety and self-stimulatory conduct, and decreased perception of pain [2, 3]. In addition, more than 25% of the PMS cases present epileptic symptoms, such as grand mal, focal and absence seizures [2, 4, 5]. Of note numerous studies have linked the SHANK3 gene with ASD [6–8], and it is estimated that mutations or deletions in the SHANK3 gene account for approximately 1% of all ASD cases [8].

Despite extensive research on the synaptic functions of Shank3, the lack of effective therapies for PMS and ASD underscores the critical need to identify the underlying pathogenic mechanisms of these disorders. Brain activity relies on the interplay between excitation (*E*) and inhibition (*I*) via synaptic communication between glutamatergic and GABAergic neuron. Although GABAergic neurons comprise only about 20% of cortical neurons, they have an important role in modulating cortical functions and plasticity; indeed, GABAergic neurons are essential for synchronizing network activity and maintaining the excitatory and inhibitory dynamics [9–12]. In addition to their role in modulating cortical functions and plasticity, GABAergic neurons are also critical for the maturation of neural circuits [13] and are implicated in developmental processes, such as regulation of neuronal proliferation and migration [14, 15]. Therefore, loss or dysfunction of these neurons has been implicated in several neurological disorders including epilepsy, schizophrenia, and autism [12, 16–18]. Interestingly, preferential deletion of *Shank3* in GABAergic cortical neurons has been associated with pyramidal neuron hyperexcitability and sensory hypersensitivity [19]. Among GABAergic neurons, parvalbumin-positive (PV) neurons have been implicated in the etiology of neuropsychiatric disorders based on a growing body of evidence [16, 20].

The PV neurons, which constitute 50% of GABAergic cells, include fast-spiking basket cells and chandelier cells. Fast-spiking PV neurons are capable of generating action potentials at high-frequency mediating fast and precise inhibition of target neurons. These cells are involved in important functions, including the regulation of plasticity, the control of gain control in the cortex and the establishment and maintenance of cortical rhythms [9, 19, 21, 22]. The activation of PV neurons is particularly critical for the generations of gamma frequency oscillations (30–80 Hz), which are a key feature of pyramidal cell synchronization and are implicated in higher brain functions, such as sensory perception, motor behavior and memory formation [13, 22–24]. Shank3 is expressed in PV GABAergic neurons, and its absence results in reduced PV expression in the striatum of Shank3 KO mice [25]. Additionally, null mutant *Shank3* exhibits delayed circuit maturation during development particularly in perisomatic PV neuronal circuit function [26].

In this study, we recorded local field potentials and visual evoked responses in the visual cortex of Shank3∆11^−/−^ (Shank3 KO) mice [27] and observed that responses to visual stimuli had a limited dynamic range likely resulting from impaired gain control in *Shank3* KO compared with Wt mice. Since gain adaptation is due to the feedback from PV interneurons [28], we hypothesized that this was due to reduced inhibition. Our hypothesis was supported by the fact that gain adaptation was restored following the potentiation of GABA_A_ receptor activity with Midazolam, suggesting a role for Shank3 in the modulation of GABAergic neurons.

In order to investigate whether deletion of *Shank3* in PV-expressing neurons leads to behavioral deficits in mice, we generated a new mouse model by crossing *Shank3* floxed mice [29] with Pv-Cre mice. Our findings indicate that the heterozygous deletion of *Shank3* specifically in PV neurons is sufficient to induce specific behavioral alterations, including repetitive behaviors, motor problems and memory impairments. Additionally, LFP recordings of Pv-Cre +/− *Shank3*^Wt/Fl^ mice revealed that selective deletion of *Shank3* in PV neurons caused a severe phenotype that led to cortical hyperexcitability. Finally, we demonstrated that the potentiation of GABA_A_ receptor activity may represent a possible therapeutic strategy to ameliorate some of the behavioral symptoms caused by Shank3 deletion.

## Methods

### Mice

Pv-Cre^+/−^ *Shank3*^Wt/Fl^ mice were generated by breeding *Shank3* floxed mice (kindly provided by Tobias Böeckers, from the Institute for Anatomy and Cell Biology of Ulm University, Germany) with Pv-Cre^+/−^ mice (*PValb*^*tm1(cre)Arbr*^, The Jackson laboratory). To visualize PV^+^ interneurons, we generated Pv-Cre^+/−^ TdTomato^Fl/−^ *Shank3*^Fl/Wt^ mice using TdTomato^+/+^ knock-in mice (*Gt(ROSA)26Sor*^*tm14(CAG−tdTomato)Hze*^, The Jackson laboratory). The *Shank3Δ11*^−/−^ mice were generated as previously described by Schemeisser et al., 2012 [27] and re-derived in a C57BL/6 background (Charles River Laboratories, Calco, Italy). These mice have already been characterized by [30]. All experiments conducted for this project involved mice that were housed in an animal facility maintained at a constant temperature (22 +/− 1 °C) and humidity (50%), with a 12-h light/dark cycle, and provided with ad libitum access to food and water. The experiments were performed in accordance with the guidelines approved by the European Communities Council and the Italian Ministry of Health (Rome, Italy) for the ethical use of laboratory animals in research. All possible efforts were taken to minimize the number of mice used and reduce their suffering. Animals from both sexes were included in all experiments, and subsequently, the data were combined, as no statistical differences were observed between the two groups.

### Mice genotyping

All primers were provided by Thermo Fisher; the REDExtract-N-Amp PCR Reaction Mix™

(Sigma-Aldrich) and the MyFi™ DNA Polymerase (Meridian Bioscience) were used for the polymerase reaction. PCR genotyping was performed using the following sets of oligonucleotide primers: parvalbumin for Wt allele forward 5′-CAGAGCAGGCATGGTGACTA-3′, for Wt allele reverse 5′-AGT ACCAAGCAGGCAGGAGA-3′, for mutant allele forward 5′-GCGGTCTGGCAGTAAAAACTATC-3′, for mutant allele reverse 5′-GTGAAACAGCATTGCTGTCACTT-3′; Shank3 floxed forward 5′-GTCTCTGTGGTTGGGGTGTC-3′, reverse 5′-CAGTGAAGAAGCCCCAGAAG-3′ for both Wt and mutant allele; TdTomato for Wt allele forward 5′- AAGGGAGCTGCAGTGGAGTA-3′, for Wt allele reverse 5′-CCGAAAATCTGTGGGAAGTC-3′, for mutant allele forward 5′- CTGTTCCTGTACGGCATGG -3′, for mutant allele reverse 5′- GGCATTAAAGCAGCGTATCC -3′; Shank3Δ11^−/−^ for Wt allele forward 5′-CAAGTTCATCGCTGTGAAGG-3′, for mutant allele forward 5′-CCTCTAGGCCTGCTAGCTGTT-3′, reverse 5′-AAGAAGCCCCAGAAGTGACA-3′ for both Wt and mutant allele.

### Surgical procedures

Mice were anesthetized with a solution of 20% urethane in physiological solution (0.9% NaCl) with a final dose of 0.8 ml/hg ([23, 24, 31, 32]; 1.6 g/kg). The depth of anesthesia was evaluated by monitoring the pinch withdrawal reflex and other physical signs (respiratory and heart rate). Additional doses (10% of initial dose) were intraperitoneally administered to maintain the level of anesthesia if necessary. The brain surface was routinely moistened with the addition of artificial cerebrospinal fluid (ACSF) at body temperature. The head of the mouse was fixed in a stereotaxic apparatus, and a portion of the skull overlying the visual cortex (0.0 mm anteroposterior and 2.7 mm lateral to the lambda suture) was drilled on the right hemisphere. A chamber was created around the craniotomy applying a thin layer of dental cement (Vertex Dental). The mouse was placed with its left eye in front of the monitor at 30 cm, oriented 45*°* with respect to the medial sagittal plane of the animal. Local field potentials (LFP) were recorded with glass micropipettes (impedance ~ 2 M*Ω*), filled with ACSF solution and connected to the amplifier head stage with an Ag/AgCl electrode. The microelectrode was positioned into the visual cortex at a depth of 250–300 μm (II/III layer) with a motorized micromanipulator. A common reference Ag/AgCl electrode was placed on the cortical surface in the ACSF cortical bath. Electrophysiological signal was amplified 1000-fold with a multichannel differential amplifier (EXT-02F, NPI), band-pass-filtered (0.1–1000 Hz) and sampled at 2 kHz with 16-bit precision by a National Instruments (NI-usb6251) ADC board controlled by custom-made LabView software. Line frequency 50 Hz noise was removed by means of a linear noise eliminator (Humbug, Quest Scientific). LFP recording lasted around 45 min, after which some animals have been superfused over the visual cortex with Midazolam (5 mg/ml in ACSF; 200 µl volume; BI- Istituto Biochimico Italiano Giovanni Lorenzini S.p.A.) or its vehicle (ACSF). After administration, the mouse was left resting, in the dark, for 1 h. Then, a second round of LFP recording was performed. Visual stimuli were provided via an LCD monitor (120 Hz frame rate, Asus). The stimuli were generated with a custom-made MATLAB program based on Psychophysics Toolbox 3 [33]. The stimulus consisted in a black and white contrast-reversing checkerboard (0.5 Hz; 0.04 cycles/degree). The checkerboard contrast was changed between different traces. The contrast values were calculated using the formula:

$$K = \frac{{\frac{L}{l} - \frac{{L_{\min } }}{{l_{\min } }}}}{{\frac{{L_{\max } }}{{l_{\max } }} - \frac{{L_{\min } }}{{l_{\min } }}}}*100\% ,$$

where *K* indicates contrast; *L* and *l* indicate, respectively, the maximum and minimum luminance of the stimulus; *L*_max_ and *l*_max_ indicate, respectively, the maximum and minimum luminance of the highest luminance stimulus and *L*_min_ and *l*_min_ the maximum and minimum luminance of the lowest luminance stimulus. Screen luminance was measured using a photometer (Konica Minolta). Six contrast values were used from maximum to minimum and randomly alternated (100%, 28%, 5.7%, 2.3%, 1.8% and 0%).

### Up states (US) and down states (DS) detection

Analysis of slow-wave oscillations was performed computing the short-time root mean square (RMS) power of the data band passed in the gamma band (25–80 Hz). The time course of the RMS power was computed on a moving window of 250 ms width and 150 ms overlap. The distribution of the logarithm of the time-resolved gamma power was bimodal, reflecting high gamma activity during USs and low gamma activity during down states. This distribution was then fitted with a double Gaussian function and the threshold for the discrimination of USs was chosen, minimizing false positives and false negatives, by computing the ROC curve. A cutoff in the minimum up (down) states duration was set to 100 ms, and up (down) state shorter than the cut-off was assigned to the ongoing down (up) state (see [32] for details).

### Spectral analysis

Spectrograms have been computed using the *mtspecgramc* function of the Chronux toolbox, with an overlapping Hammer window of 150 ms width and 50 ms overlap. Multitaper power spectra have been calculated on 90-s traces recorded from anesthetized mice with no visual stimulation, using the *mtspectrumc* function of the Chronux toolbox [25].

### Analysis of visual evoked responses

The visual evoked potential (VEP) of each mouse was calculated as the average of 120–180 trials. The peak amplitude was measured in a window from 90 to 500 ms after the stimulus, with respect to a baseline level computed in the 30–80 ms window. Contrast transfer functions obtained for every mouse have been fitted with a Michaelis Menten function:$A(k) =\frac{{A}_{\mathrm{max}}*k}{{k}_{H}+k}$, where A is the response amplitude, k is contrast, A_max_ is the response at "saturating" contrast levels and k_H_ is the contrast at which the amplitude is equal to half M. The VEP slope was calculated as the angular coefficient of a line fitting the VEP in a window of 80 ms centered on the half-peak value. Gamma band power (25–80 Hz) during the response was calculated for every trial as the RMS power of the filtered trace in two different time windows. The windows were 30–80 ms and 90–500 ms after the stimulus, called, respectively, "baseline" and "response"; then the base-10 logarithm of the ratio between response and baseline gamma power has been calculated and averaged among trials.

### Statistical analysis for electrophysiology

In the figure legends are reported the number of replicates (n) and the statistical analyses used for every experiment. Based on the number of condition and the number of groups in the comparison, appropriate statistical tests were used to analyze the data. First was analyzed the data distribution by the Shapiro–Wilk normality test. For the comparisons, parametric tests including Student *t*-tests and one-way and two-way analysis of variance (ANOVA) with appropriate post hoc test were used if the data were normally distributed; nonparametric test including the Mann–Whitney test and Kruskal–Wallis test with appropriate post hoc test were used for data with non-normal distribution. Results are presented as box plots presenting the 1^st^ and 3^rd^ quartile, the median and the mean. Since each data point represents an experimental mouse, the total number of data points is limited and the box plots do not include whiskers, but all data points are presented, thus allowing a proper understanding of scattering and range. Statistics were computed as described in the figure legends.

All electrophysiological data analysis was performed with custom-made software suite written in MATLAB (https://github.com/DidiLamers/PCDH19_ZebraExplore) [34].

### Immunohistochemistry

Mice were anesthetized and perfused transcardially with sucrose 5% and paraformaldehyde 4%. Brains were removed and post-fixed overnight in paraformaldehyde 4%. The day after three washes with PBS were performed and brains were incubated for at least eight hours in 30% sucrose. Finally, brains were included in cryomolds with Tissue-Tek OCT compound (Bio-Optica) and put at − 80 °C until cryostat sectioning. Brains were cut with a cryostat and 20 μm-thick coronal slices were collected on polysine microscope adhesion slides (ThermoFisher). Slices were incubated first in blocking solution (3%BSA, 10% goat serum, 0.4% Triton-X-100, diluted in PBS) for at least 30 min and then they were incubated with primary antibodies overnight at 4 °C. Subsequently, three wash (10 min each) with PBS were performed and brain slices were incubated with fluorophore-conjugated secondary antibodies (Jackson ImmunoResearch Laboratories) for 1 h at room temperature. After the antibody incubation, brain slices were washed and 4',6diamidino-2-phenylindole (DAPI) staining (ThermoFisher) was carried out (DAPI diluted in PBS to a final concentration of 0.5 μg/ml). Finally, another washing step is performed before mounting the coverslips with Mounting Medium (Vecta Shield). Primary antibodies used were: anti-Parvalbumin (Swant, GP72), Shank3 (homemade SHANK3rb [35]).

### Microscopic analysis

Brain areas were selected according to the mouse brain atlas of Paxinos and Franklin (Paxinos & Franklin, 2008) from each of the following brain regions at the following bregma coordinates: prefrontal cortex (from 2.4 to 1.7 mm), visual cortex (from − 2.06 to − 2.70 mm), hippocampus (from − 1.06 to − 2.2 mm). Confocal images were obtained using LSM800 confocal microscope (Carl Zeiss) with Zeiss 10 × objective at a resolution of 1024 × 1024 pixels, under the same condition across different mice and brain slices. PV-positive cells were counted in the bilateral areas of each section using ImageJ software.

For Shank3 staining, imaging was performed on a laser-scanning microscope (Leica DMi8) with 63 × oil DIC immersion objective (ACS Apo, NA 1.3) with xy resolution 159 nm and z resolution 345 nm with a stack size of 1.73 µm. Deconvolution of the images was performed using Huygens Essentials 22.04 software (Scientific Volume Imaging B.V.). Subsequently, an analysis of the images was performed with surface and spot tool with Imaris 9.9 Software (Oxford Instruments). Parvalbumin-positive neurons within a specific region of interest (ROI) were selected with the surface tool by tdTomato signal. With the spot tool Shank3-positive spots close to the surfaces (If < or equal to 0.5 µm distance) were determined. Number of spots close to surface within one ROI was normalized on the surface volume within the ROI. Two sections (3 ROIs per section) were analyzed for each hemisphere per animals.

### Biochemistry

Cortex and hippocampi were dissected from mouse brain of both sexes. Tissues were homogenized in buffer containing 10 mM Hepes pH 7.4, 2 mM EDTA, protease inhibitors (Roche), and phosphatase inhibitors (Sigma, P8340). Samples were centrifuged at 500 × g for 5 min at 4 °C. Resulting supernatants were centrifuged at 10,000 × g for 15 min at 4 °C. After the centrifugation, pellets were resuspended in buffer composed by 50 mM Hepes pH 7.4, 2 mM EDTA, 2 mM EGTA, 1% triton-X-100 (Sigma), protease inhibitors, and phosphatase inhibitors and centrifuged at 20,000 × g for 80 min at 4 °C. Finally, pellets were resuspended in buffer containing 50 mM Tris pH 9, 1% sodium deoxicholate (Sigma, D6750). Samples were quantified by BCA protein assay (EuroClone) to assess protein concentration. Equal amounts of each sample were separated using SDS-PAGE and subsequently blotted on nitrocellulose membranes using the Trans- Blot Turbo System (BioRad). Membranes were washed in Tris-buffered saline-Tween (TBS-T) (200 mM Tris pH 7.4, 1.5 M NaCl (both Sigma-Aldrich) and 0.1% Tween 20% (BioRad). After 1 h blocking at room temperature with 5% bovine serum albumin or milk in TBS-T, membranes were incubated overnight at 4 °C with primary antibody (Shank3, Santa Cruz Biotechnology, cat. H-160; GABA-A-R-alpha1, Neuromab, cat. 75136; Parvalbumin, Swant, cat. GP72; β3-tubulin, Sigma, cat. T8578). Membranes were washed in TBS-T and then incubated with HRP-conjugated secondary antibodies (Jackson ImmunoResearch) for 1 h at room temperature. After three washes, chemiluminescence was induced using an ECL Western Blotting Substrate kit and further detected using a ChemiDoc XRS + machine. All signals were quantified using ImageLab software and normalized against the values of the respective signal for βIII-tubulin.

### Behavioral analysis

Mice of P60-P90 were used for behavioral experiments. Animals were housed in groups of four or five individuals. All of the tests were conducted during the light portion of the cycle. The sample size of animals required for behavioral analysis was estimated using the G*Power 3.1 statistical analysis program, setting a 95% confidence limit (*α* err. prob. = 0.05) and a power of 80%. The effect size was calculated using the formula Δ/SD (Δ = difference between the groups; SD = standard deviation). Means and standard deviations were estimated based on previous experiments in the laboratory. Based on these parameters, we determined that a sample size of at least 5 animals was necessary to detect any differences between groups.

### Repetitive self-grooming test

The spontaneous self-grooming behavior was assayed as described in [36]. Single mouse was placed into a standard cylinder (46 × 23.5 × 20 cm). Cylinders were empty to eliminate digging in the bedding, which is a potentially competing behavior. The room was illuminated at about 40 lx. A front-mounted CC TV camera (Security Cameras Direct) was placed at circa 1 m from the cages to record the sessions. Sessions were video-taped for 20 min. The first 10 min of habituation was not scored. Cumulative time spent grooming all the body regions during the second 10 min of the test session was measured.

### Novel object recognition test

The novel object recognition test was performed in an open plastic arena (60 × 50 × 30 cm). The test had three phases: the habituation on the first day, when mice were accustomed to the test arena for 10 min; the familiarization; and the novel object recognition the day after. In the familiarization phase, two identical objects were placed in the middle of the arena equidistant from the walls and from each other. Mice were placed between the two objects until it had completed 30 s of cumulative object exploration (20 min cut-off). Experimenter measured the time mice was within approximately 1 cm of an object with its nose toward the object. Climbing the object or pointing the nose toward ceiling near the object were not considered exploring behaviors. After familiarization, mice returned to the home cage until they were tested for novel recognition after 5 min, 120 min and 24 h. In the novel recognition phase, a novel object (never seen before) took the place of one of the two familiars. Scoring of object recognition was performed as during the familiarization phase. For each mouse, the role (familiar or new object) as well as the relative position of the two objects were randomly permuted. The objects used for the test were white plastic cylinders and colored plastic Lego stacks of different shapes. The arena was cleaned with 70% ethanol after each trial. Performance was analyzed by calculating the discrimination index (*N* − *F*/*N* + F), where *N* = the time spent exploring the novel object, and *F* = the time spent exploring the familiar object.

### Spatial object recognition test

Spatial object recognition test was performed in an arena according to [30], with minor modifications that consisted in an opaque white Plexiglass cage (58 × 50 × 43 cm) that was dimly lit from above (27 lx) and two visual cues that were placed above two adjacent walls. The visual cues consisted of a black and white stripped pattern (21 × 19.5 cm) that was affixed to the center of the northern wall and a black and gray checkered pattern (26.5 × 20 cm) that was placed in the center of the western wall. Objects were placed across the visual cues. Mice were habituated to the arena for 10 min the day before the test. Twenty-four hours later, it is initially performed a training session where mice were allowed to familiarize with two different objects. The experimenter measured the time spent in sniffing both objects until the mouse completed 30 s in exploring objects (cut-off 20 min). Exploring behavior was defined as mouse having its nose directed toward the object and within approximately 1 cm of the object [37]; climbing or sitting were not considered exploration behaviors. After 5 min, 120 min and 24 h, mice were allowed to re-explore the cage where one object was moved in a new position. Between two sessions, mice returned to their home cage. Cage and object were carefully cleaned with 70% ethanol before and after all behavioral procedures. Performance was analyzed by calculating a discrimination index (*N* − *F*/*N* + *F*), where *N* = the time spent exploring the moved object during the test, and *F* = the time spent exploring the unmoved object during the test.

### Balance beam walking test

The beam apparatus consisted of 1 m beams with a flat surface of 12 mm or 6 mm width resting 50 cm above the table top on two poles. A black box was placed at the end of the beam as the finish point. Nesting material from home cages was placed in the black box to attract the mouse to the finish point. A lamp (with 60-W light bulb) was used to shine light above the start point and served as an aversive stimulus. A video camera was set on a tripod to record the performance. On training days, each mouse crossed the 12 mm beam 3 times and then the 6 mm beam 3 times. The time required to cross to the escape box at the other end (80 cm away) was measured with a stopwatch. The stopwatch started when the nose of the mouse began to cross the beam, and stopped when the animal reaches the escape box. Once the mice are in the safe box, they are allowed some time (~ 15 secs) to rest there. Before the next trial the mice rest for 10 min in their home cages between training sessions on the two beams. On the test day, times to cross each beam were recorded. Two successful trials in which the mouse did not stall on the beam are averaged. The beams and box were cleaned with towels soaked with 70% ethanol and then water before the next beam was placed on the apparatus.

### Rotarod test

The rotarod apparatus (Ugo Basile, Biological Research Apparatus, Varese, Italy) was used to measure fore and hindlimb motor coordination and balance. During the training period, each mouse was placed on the rotarod at a constant speed (12 and 32 rpm) for a maximum of 120 s, and the latency to fall off the rotarod within this time period was recorded. Mice received four trials per day for 4 straight days. The fourth trial of each day was evaluated for statistical analysis.

### Pole test

In the pole test, the mouse was placed on a vertical wire-mesh pole (90 cm length, 1 cm diameter) with its head facing upwards. Mice were habituated to descend the pole in 2 trials per day for 2 days. On test day (third day) mice were subjected to 5 trials: the total time taken to turn the body and to descend the pole was recorded according to [38]. A cutoff of 60 s was given. Data were shown as mean of 5 trials evaluated during the test day.

### Wire hanging test

The limb force was tested by positioning a mouse on the top of a wire cage lid (19 × 29 cm) that was turned upside down at approximately 25 cm above a surface with the bedding material. The grip of the mouse was ensured by gently waving three times before rotating the lid as described in [39]. The latency to fall onto the bedding was recorded over a maximum period of 300 s.

### Sociability and social novelty test

The sociability tests were performing in an apparatus called three-chambered box. It is formed by a rectangular transparent polycarbonate apparatus with three-chamber (width = 42.5 cm, height = 22.2 cm, central chamber length = 17.8 cm, and side chamber lengths = 19.1 cm) as previously described in [36]. The three-chamber box was illuminated by diffuse incandescent lighting (15 lx), and a video camera was located directly over the center of the open field.

First, the test includes a habituation phase during which the mouse was placed in the middle chamber free to explore all the compartments for 10 min. After this period, novel conspecific mouse, that had no previous interaction with the test mouse, was placed in one of the side chambers. The doors were unblocked and the subject mouse was given the possibility to explore for 10 min either an empty chamber or a chamber containing the stranger mouse. In the 10-min trial, the duration of contact with the counterpart was recorded. Immediately after sociability test, without cleaning the apparatus, it was performed the social novelty test putting an unfamiliar mouse in the empty wire cage. Time spent in each chamber was recorded. The data were expressed in sociability index (SI) and social novelty preference index (SNI) as follows: SI = (time exploring novel mouse 1 – time exploring empty cage) / (time exploring novel mouse 1 + time exploring empty cage) and SNI = (time exploring novel mouse 2 – time exploring familiar mouse) / (time exploring novel mouse 2 + time exploring familiar mouse).

### Pharmacological treatment

Pv-Cre^+/−^ *Shank3*^Wt/Wt^, Pv-Cre^+/−^ *Shank3*^Fl/Wt^, *Shank3* Wt and *Shank3* KO mice were treated with a selective positive allosteric modulator of GABA_A_ receptors: Ganaxolone. Ganaxolone (Tocris Cat. No. 2531) was resuspended in ethanol and betaciclodextrin (Sigma-Aldrich 332607). Mice received an intraperitoneal injection of ganaxolone (5 mg/Kg) or vehicle 30 min before each behavioral test.

## Results

### Gain control is impaired in Shank3 KO mice

**Fig. 1:**
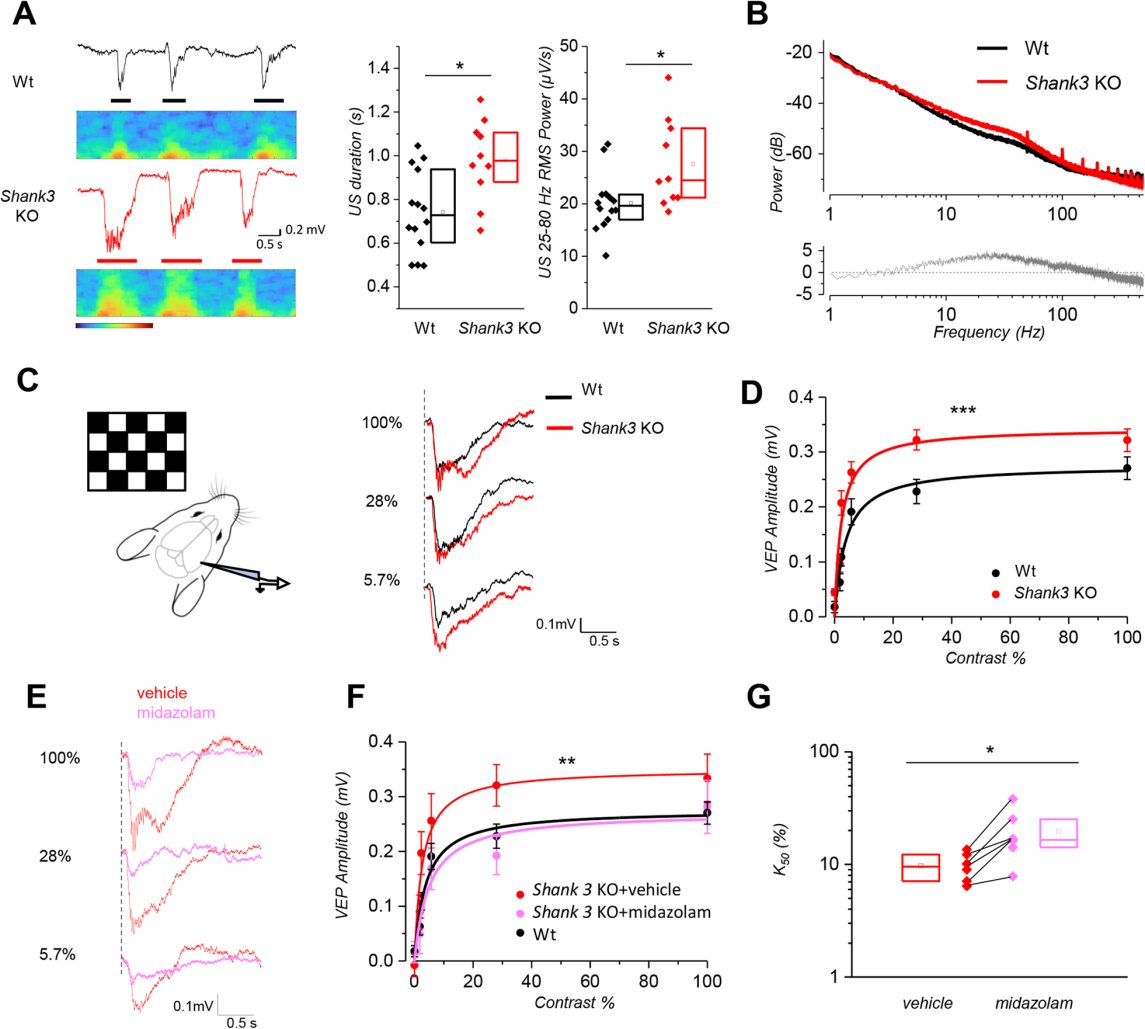
*Shank3* KO mice are hyperexcitable and show an altered gain control. **A** *left*: Representative electrophysiological traces of up and down states in Wt (*black*, top) and *Shank3* KO (*red,* bottom) anesthetized mice with the respective spectrograms. USs are indicated with a bar. Scale bar 0.2 mV, 0.5 s. Color map for spectrograms: -80, -20 dB. *Middle:* Analysis of slow-wave activity in the same experimental groups shows increased duration of USs in *Shank3* KO mice (Wt, *N* = 14 mice, *n* = 40 traces; *Shank3* KO, *N* = 11 mice, *n* = 31 traces; Mann–Whitney Test, *p < 0.05), where up and down states were identified automatically by selecting power in gamma band. *Right:* US 25-80 Hz RMS Power is significantly different (Mann–Whitney Test, *p < 0.05). **B** *top*: Mean power spectra of LFP in anesthetized mice. Lines indicate the mean power spectra and the shaded areas indicate the SEM (*N* = 14 Wt, *N* = 11 *Shank3* KO). *Bottom*, difference between *Shank3* KO and Wt average power spectra. *Shank3* KO mice are not openly epileptic; however, spectral analysis of the resting state under urethane anesthesia shows increased spectral power at all frequencies between a few Hz to about 100 Hz; in particular, there is a diffused increase in the beta (10–25 Hz) and gamma frequency band (25-80 Hz) (Mann–Whitney rank test over frequency window from 10-20 Hz to 90-100 Hz; ***p < 0.001). The two spectral distributions are not different below 2 Hz. **C** Experimental paradigm and exemplificative VEPs to alternating checkerboards. Dotted lines indicate the checkerboard reversal. **D** Contrast sensitivity curve is significantly different in *Shank3* KO (*N* = 17) *versus* Wt mice (*N* = 17) (two-way ANOVA, Holm-Sidak test; ***p < 0.001), with *Shank*3 KO mice showing fast saturation of the response curve at lower contrast value. The continuous lines here and in panel F are Michaelis–Menten fits to the data. **E** Exemplificative waveforms of responses to alternating checkerboards (red, *Shank3* KO; magenta, *Shank3* KO after midazolam superfusion over the cortex). Scale bars: 0.1 mV, 0.5 s. **F** Contrast sensitivity curve is significantly different in *Shank3* KO treated with vehicle (*N* = 6) *versus Shank3* KO + Midazolam mice (*N* = 6) and it is evident a rescue to control situation after superfusion with Midazolam (Wt, *N* = 17; 5 mg/ml; two-way ANOVA; **p = 0.003). **G** Analysis of contrast sensitivity curves, as quantified by the Michaelis–Menten fittings (Wilcoxon Signed Ranks Test; * p = 0.035)

As the visual cortex serves as a prototypical model for studying changes in brain circuitry associated with various forms of ASD [40–43], we decided to analyze sensory processing in the visual cortex in *Shank3*Δ11^−/−^ [27] (referred as *Shank3* KO) mice by conducting local field potential (LFP) recordings. The mice were anesthetized with urethane to induce a slow-wave-like sleep as in [32, 44]. During this state, the brain cortex exhibited oscillations between states of very low neuronal activity (known as down states, DS) and states of high neuronal activity (known as up states, US). Our results demonstrate that USs in *Shank3* KO mice had a longer duration and a higher gamma power (25–80 Hz) compared to controls. However, there were no significant differences in the frequency or median amplitude of the up states between the two groups (Fig. 1A and Additional file 1: Fig. 1). This result is consistent with an enhancement of the spectral power for the frequency band 4–100 Hz, as represented in Fig. 1B, and suggests a reduction of the inhibitory drive. To further investigate the role of *Shank3* in regulating inhibition, we measured visual contrast gain control, which is heavily reliant on the inhibitory feedback of fast-spiking interneurons on pyramidal neurons. This feedback is essential for adapting the limited dynamic range of pyramidal cell firing to the large dynamic range of sensory stimuli [45, 46]. We recorded local field potentials (LFP) in response to a checkerboard contrast-reversal stimulus and measured the amplitude of the visual evoked potentials (VEPs) in relation to contrast levels (Fig. 1C). Our findings indicate that the response of *Shank3* KO mice reached a saturation level for lower contrast values, compared to the wild-type mice (Fig. 1D). The gain of the visual response was estimated for each mouse by fitting the contrast curves with a Michaelis–Menten function. The distribution of the two parameters (A_M_, asymptotic response amplitude and K_50_, half-saturating contrast) was compared between *Shank3* KO and Wt mice (Additional file 1: Fig. 2A–C). We found that K_50_ was lower in the *Shank3* KO compared to Wt mice, indicating the presence of saturation at a lower contrast and increased contrast gain in *Shank3* KO. Additionally, the recorded VEPs were steeper in *Shank3* KO mice compared to Wt mice, as represented in the analysis of the slope (Additional file 1: Fig. 2C). Spectrograms of the VEP recordings show a power increase during visual response in *Shank3* KO mice (Additional file 1: Fig. 2D–E). We quantified the power increase in the gamma spectral band both during response and in presence of a blank stimulus as a function of the stimulus contrast (Additional file 1: Fig. 2E). These curves appear to be similar to those obtained when measuring VEP amplitudes (Fig. 1D), confirming that contrast gain is greater in the *Shank3* KO mice. The increased gain we measured in response to visual stimuli is suggestive of an impairment of inhibition from fast-spiking PV interneurons, which are responsible for gain control in the visual cortex [45]. Therefore, our data suggests that the major cause of the alterations in the visual response is due to inhibitory impairment. To test this hypothesis, we measured contrast gain in *Shank3* KO mice before and after superfusion with the GABA_A_ receptor agonist midazolam (5 mg/ml). As shown in Fig. 1E–G, pharmacological activation of GABA_A_ receptor was sufficient to rescue gain control in *Shank3* KO mice compared to *Shank3* KO mice treated with vehicle only (ACSF)**.** Midazolam superfusion per se acts on resting activity reducing the high excitability apparent in *Shank3* KO mice as shown in Additional file 1: Fig. 3A–D, with no differences in US duration, frequency and amplitude. Given that Shank3 is localized on excitatory synapses only, we can rule out that the inhibition impairment originates in decreased number or strength of inhibitory synapses. Far more likely is that the excitatory drive on PV interneurons is defective, leading to reduced recruitment of PV interneurons and to impaired excitation/inhibition balance.

**Fig. 2:**
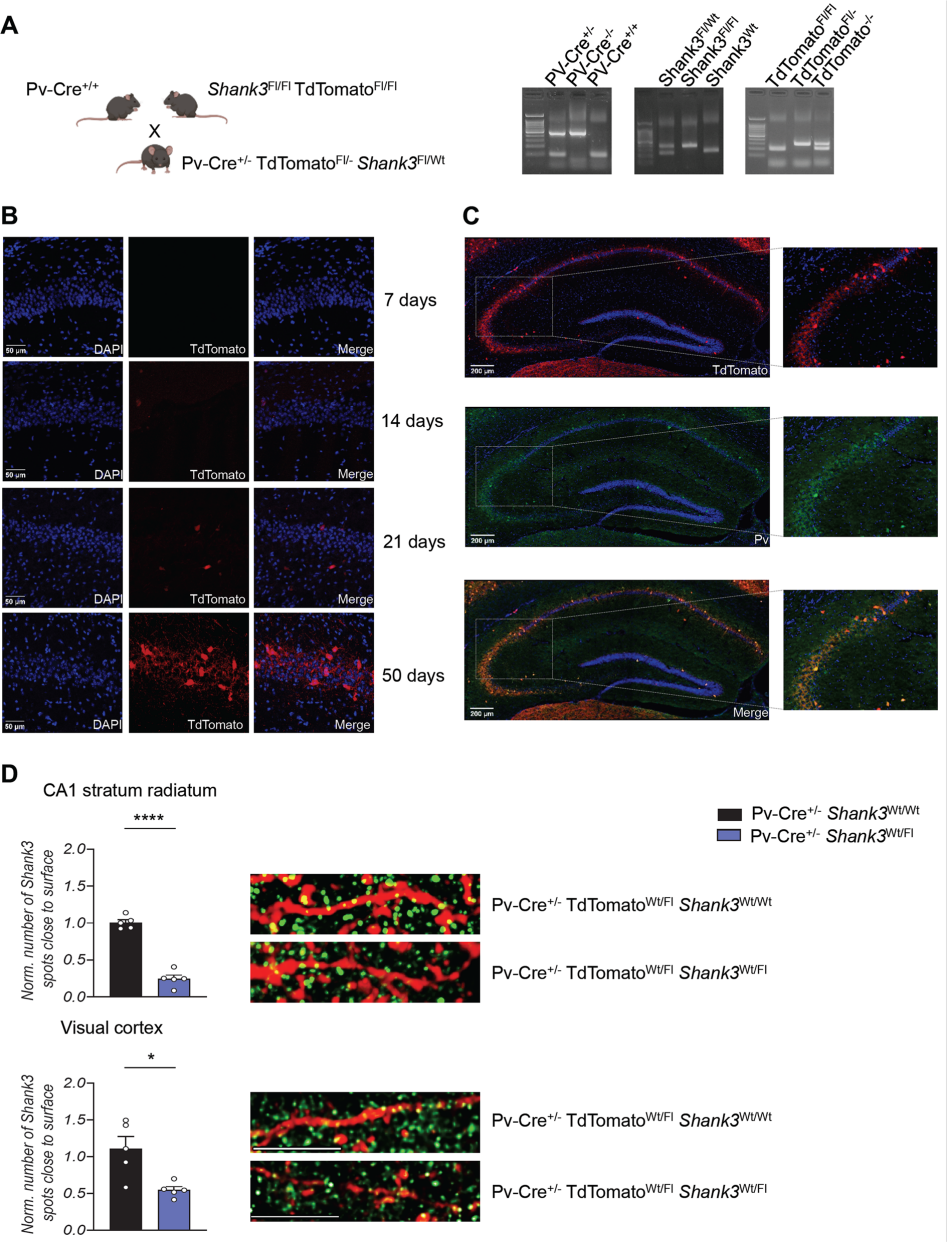
Generation of mouse model and analysis of Parvalbumin expression. **A** Right, schematic strategy for the generation of Pv-Cre^+/−^ TdTomato^Fl/−^ *Shank3*^Fl/Wt^ mice that express the gene reporter TdTomato specifically in PV-positive cells. Left, representative images of PCR genotype analysis. **B** Hippocampal fluorescence images of Pv-Cre^+/−^ TdTomato^Fl/−^ *Shank3*^Wt/Wt^ mice at different ages. Parvalbumin expression (red) begins from 14 days after birth. **C** Representative images of Pv (green) and TdTomato (red) colocalization in hippocampal slice. **D** Quantification (left) and representative images (right) of Shank3 expression (green) in PV-positive neurons in CA1 stratum radiatum and visual cortex of Pv-Cre ^+/−^ Shank3^Wt/Wt^ and Pv-Cre ^+/−^ Shank3^Wt/Fl^ mice**.** Experiment in the CA1 stratum radiatum was analyzed by unpaired, two-tailed Student’s *t*-test; Visual cortex was analyzed by unpaired, two-tailed Student’s *t*-test with Welch’s correction; *n* = 5 Pv-Cre ^+/−^ Shank3^Wt/Wt^, *n* = 5 Pv-Cre ^+/−^ Shank3^Fl/Wt^; *p < 0.05; ****p < 0.0001. CA1 = Cornu Ammonis-1. Scale bar = 10 µm

**Fig. 3:**
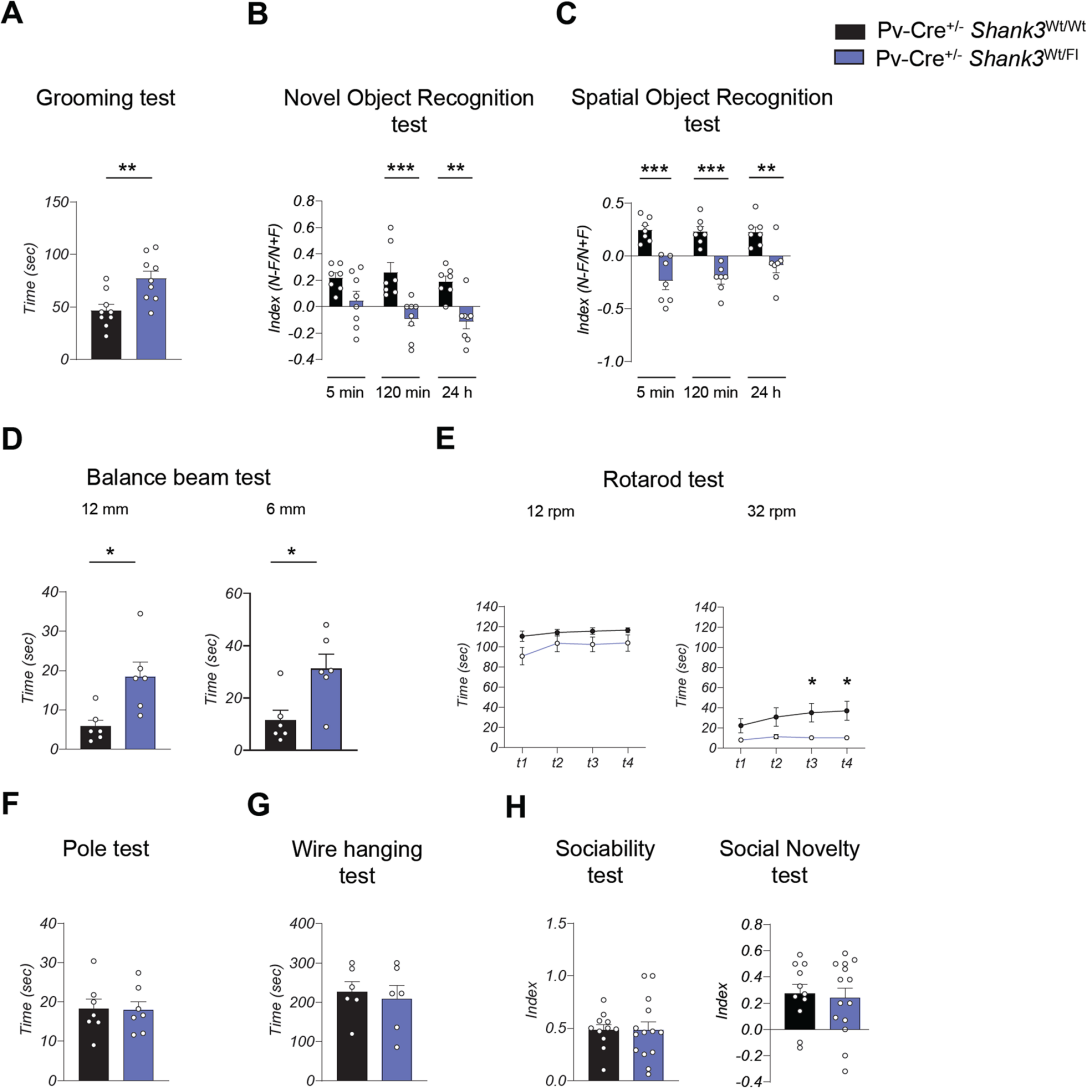
*Shank3* haploinsufficiency specifically in inhibitory PV neurons is sufficient to induce behavioral deficits. **A** Increased repetitive grooming behavior in Pv-Cre^+/−^ *Shank3*^Fl/Wt^ compared to Pv-Cre^+/−^ *Shank3*^Wt/Wt^ mice. Grooming time was analyzed by unpaired, two-tailed Student’s *t*-test; *n* = 9 for each group; **p < 0.01. **B** Pv-Cre^+/−^ *Shank3*^Fl/Wt^ mice show an impairment in the novel object recognition test. Novel object recognition at 5 min was analyzed by unpaired, two-tailed Student’s *t*-test; novel object recognition at 120 min was analyzed by two-tailed Mann–Whitney test; Novel object recognition at 24 h was analyzed by unpaired, two-tailed Student’s *t*-test; *n* = 7 Pv-Cre^+/−^ *Shank3*^Wt/Wt^, *n* = 8 Pv-Cre^+/−^ *Shank3*^Fl/Wt^; **p < 0.01; ***p < 0.001. **C** Spatial memory was evaluated by determining a discrimination index in the spatial object recognition test. Pv-Cre^+/−^ *Shank3*^Fl/Wt^ mice have an impairment in the discrimination index in all the time point evaluated. Spatial object recognition at 5 min, 120 min and 24 h were analyzed by unpaired, two-tailed Student’s *t*-test; *n* = 7 for each group; **p < 0.01; ***p < 0.001. **D** Pv-Cre^+/−^ *Shank3*^Fl/Wt^ mice show impaired motor coordination in the balance beam test. Time to cross the 12 mm width beam was evaluated by unpaired, two-tailed Student’s *t*-test; time to cross the 6 mm width beam was analyzed by two-tailed Mann–Whitney test; *n* = 6 for each group; *p < 0.05. **E** Pv-Cre^+/−^ *Shank3*^Fl/Wt^ are impaired in the rotarod test. Unpaired, two-tailed Student’s *t*-test with Welch’s correction was used for the statistical analysis; *n* = 16 Pv-Cre^+/−^ *Shank3*^Wt/Wt^, *n* = 17 Pv-Cre^+/−^ *Shank3*^Fl/Wt^; *p < 0.05. **F** Pole test analysis show no alteration in Pv-Cre^+/−^ *Shank3*^Fl/Wt^ mice. Data were analyzed by unpaired, two-tailed Student’s *t*-test; *n* = 6 for each group. **G** Pv-Cre^+/−^ *Shank3*^Fl/Wt^ mice show normal muscle strength. Wire hanging test was evaluated by unpaired, two-tailed Student’s *t*-test; *n* = 6 for each group. **H** Social interaction was evaluated by the three-chamber assays. Unpaired, two-tailed Student’s *t*-test was used for statistical analysis; *n* = 11 Pv-Cre^+/−^ *Shank3*^Wt/Wt^, *n* = 14 Pv-Cre^+/−^ *Shank3*^Fl/Wt^

### Preferential deletion of Shank3 in PV neurons causes specific behavioral alterations

Our results align with substantial evidence suggesting a role for PV interneurons in the etiology of neuropsychiatric disorders [16, 47, 48]. Therefore, to determine the specific contribution of Shank3 deletion in these neurons to the pathogenesis of PMS and ASD, we utilized Cre-lox technology to generate mice with selective deletion of *Shank3* in PV neurons. We first bred *Shank3* floxed mice with transgenic mice carrying TdTomato gene which can only be expressed after the excision of a *loxP*-flanked STOP cassette by Cre-mediated recombination. Subsequently, we crossed these mice with the line expressing Cre-recombinase under the PV promoter. This allowed us to easily visualize PV neurons with the deletion of *Shank3* using TdTomato. PCR analysis confirmed the genotype of these mice (Fig. 2A). Furthermore, immunofluorescence showed that TdTomato expression increased during brain development (Fig. 2B) and that cells expressing TdTomato were also positive for parvalbumin expression (Fig. 2C). We confirmed that Shank3 was downregulated in Pv-Cre^+/−^ TdTomato^Wt/fl^ Shank3^Wt/Fl^ mice compared to controls (Fig. 2D). Subsequently, we examined the impact of selective deletion of Shank3 in PV neurons on mouse behavior. Surprisingly, our analysis revealed that the deletion of a single Shank3 allele in PV neurons was sufficient to induce behavioral changes, which contrasts with the absence of cognitive deficits in Shank3 full KO mice in heterozygosity [30]. Pv-Cre^+/−^ *Shank3*^Wt/Fl^ spent significantly more time in doing grooming compared to Pv-Cre^+/−^ *Shank3*^Wt/Wt^ (referred as control mice) suggesting that *Shank3* deletion in PV neurons is sufficient to cause repetitive behavior (Fig. 3A). Memory performance was also affected by selective deletion of Shank3 in PV neurons, as Pv-Cre^+/−^ *Shank3*^Wt/Fl^ exhibited deficits in both the novel object recognition test (Fig. 3B) and the spatial object recognition test (Fig. 3C). Furthermore, Pv-Cre^+/−^ *Shank3*^Wt/Fl^ displayed impaired motor coordination taking longer to cross the beam than control mice (Fig. 3D) and displayed some motor problems, as demonstrated by the results of the rotarod test (Fig. 3E). However, they did not exhibit any impairments during the pole test (Fig. 3F) nor a reduction in muscular strength (Fig. 3G). The three-chamber test did not reveal any differences between Pv-Cre^+/−^ *Shank3*^Wt/Fl^ mice and control mice in sociability or social novelty (Fig. 3H). Social performances were also unaffected in the homozygous Pv-Cre^+/−^ *Shank3*^Fl/Fl^ mice, indicating that *Shank3* deletion only in PV neurons was insufficient to affect mice sociability (Additional file 1: Fig. 4A). Finally, we investigated whether homozygous Shank3 deletion in PV neurons exacerbates the phenotype observed in Pv-Cre^+/−^ *Shank3*^Wt/Fl^ mice. However, as demonstrated by the results of the grooming test and novel object recognition test, we observed only a slight deterioration in behavior compared to heterozygous mice, which did not appear to be significant (Additional file 1: Fig. 4B and C).

**Fig. 4:**
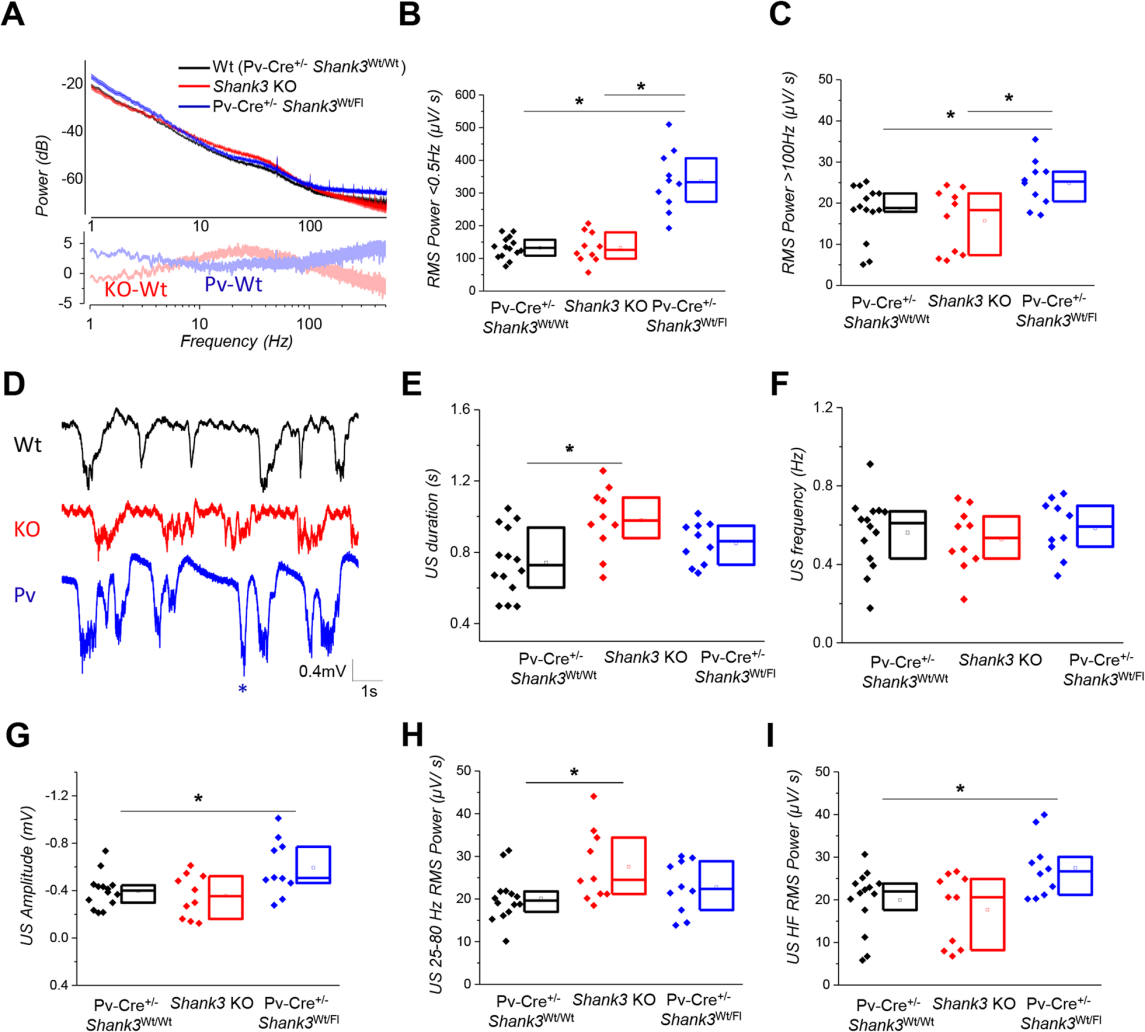
E/I balance is altered toward excitation when *Shank3* is selectively disrupted in PV neurons even if in heterozygosis. **A** Spectral power analysis of Pv-Cre^+/−^ *Shank3*^Wt/Wt^ (*N* = 14 mice), *Shank3* KO (*N *= 11 mice) and Pv-Cre^+/−^ *Shank3*^Fl/Wt^ mice (*N *= 10 mice). Spectral differences between *Shank3* KO and Pv-Cre^+/−^ *Shank3*^Wt/Wt^ (indicated as KO-Wt in light red), and Pv-Cre^+/−^ *Shank3*^Fl/Wt^ and Pv-Cre^+/−^ *Shank3*^Wt/Wt^ (indicated as Pv-Wt in light blue) show a distinct E/I behavior in the two models, even in the direction of a more epileptic phenotype. **B**, **C** Graphs show an alteration of the spectral power for power less than 0.5 Hz (**B**) or frequencies larger than 100 Hz (C) (one-way ANOVA; *p < 0.05). **D** Exemplificative waveforms of slow-wave activity in control (black), *Shank3* KO (red) and Pv-Cre^+/−^ *Shank3*^Fl/Wt^ (blue) anesthetized mice in which it appears evident the difference in US and DS modulation. * indicates a large transient suggestive of hypersynchronicity. Scale bar 0.4 mV, 1 s. **E**, **F** *Shank3* mutation in PV neurons activity doesn’t affect US duration or frequency. **G–I** *Shank3* mutation in PV neurons activity specifically affects US amplitude (**G**) and US power in HF band (**I**); one-way ANOVA, *p < 0.05), while doesn’t not affect US 25-80 Hz gamma power differently from what happens in *Shank3* KO mice (see Fig. 1A)

### Shank3 deletion in PV neurons induces cortical hyperexcitability

Next, we recorded the resting state LFP in the visual cortex of Pv-Cre^+/−^ *Shank3*^Wt/Fl^ mice under urethane anesthesia. Power spectra analysis showed that Pv-Cre^+/−^ *Shank3*^Wt/Fl^ mice had a significantly higher spectral power compared to controls at both low (< 5 Hz) and high frequencies (> 100 Hz Fig. 4A–C). Further detailed analysis of the SWA revealed that there was no significant difference in the frequency and duration of up states between the two groups (Fig. 4E, F). However, the amplitude of up states was larger in Pv-Cre^+/−^ *Shank3*^Wt/Fl^ mice, suggesting higher neuronal firing during up states in this group (Fig. 4 G). Additionally, the measured power during USs was significantly higher for Pv-Cre^+/−^ *Shank3*^Wt/Fl^ mice in HF band (> 100 Hz) (Fig. 4I). These findings are all indicative of a higher degree of excitability, suggesting that selective deletion of *Shank3* in PV neurons caused a specific excitation/inhibition (*E*/*I*) balance that leads to cortical hyperexcitability.

**Fig. 5:**
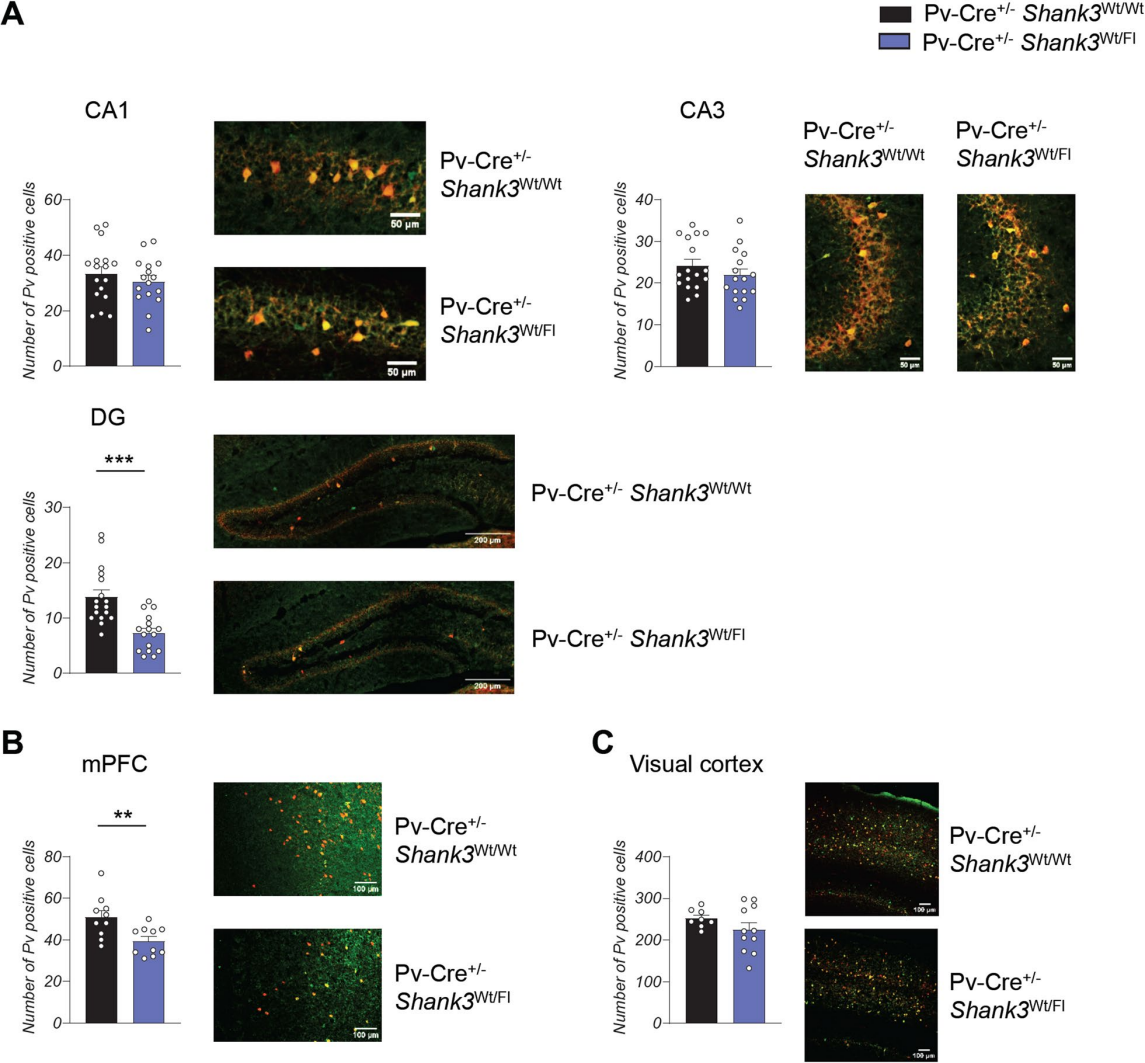
Decrease of inhibitory interneurons in Pv-Cre^+/−^ *Shank3*^Fl/Wt^ mice. **A** Quantification and representative images of the number of PV neurons in the hippocampus. Pv-Cre^+/−^ *Shank3*^Fl/Wt^ mice show a reduction of Parvalbumin interneurons in the dentate gyrus of the hippocampus. Data from the CA1 were analyzed by unpaired, two-tailed Student’s *t*-test; quantification of the CA3 and DG were analyzed by two-tailed Mann–Whitney test; *n* = 17 Pv-Cre^+/−^ *Shank3*^Wt/Wt^, *n* = 16 Pv-Cre^+/−^ *Shank3*^Fl/Wt^; *** p < 0.001; *n* = number of bilateral slides analyzed; 3 animals used for each group. CA1 = Cornu Ammonis-1; CA3 = Cornu Ammonis-3; DG = dentate gyrus. **B** Quantification and representative images of the number of PV neurons in the medial prefrontal cortex. Data were analyzed by unpaired, two-tailed Student’s *t*-test; *n* = 9 Pv-Cre^+/−^ *Shank3*^Wt/Wt^, *n* = 8 Pv-Cre^+/−^ *Shank3*^Fl/Wt^; ** p < 0.01; *n* = number of bilateral slides analyzed; 3 animals used for each group. **C** Quantification and representative images of the number of PV neurons in the visual cortex. Data were analyzed by unpaired, two-tailed Student’s *t*-test with Welch’s correction; *n* = 8 Pv-Cre ± Shank3Wt/Wt, *n* = 11 Pv-Cre ± Shank3Fl/Wt; *n* = number of bilateral slides analyzed; 3 animals used for each group

We subsequently conducted a quantitative analysis to determine the density of parvalbumin (PV) neurons in different brain regions that exhibit high levels of Shank3 expression. We conducted an analysis of the number of Tdt + and Pv + cells and the percentage of colocalization across multiple brain regions, including the mPFC, CA1, CA3, DG, and visual cortex. Our results indicate that in all brain regions examined, 77% of Tdt + cells also express Pv + , suggesting high specificity of the model. In the hippocampus of Pv-Cre^+/−^ Shank3^Wt/Fl^ mice, we observed a significant decrease in the number of PV neurons specifically in the dentate gyrus, whereas no significant difference was detected in the CA1 and CA3 regions (Fig. 5A). Similarly, in the medial prefrontal cortex (mPFC) of Pv-Cre^+/−^ Shank3^Wt/Fl^ mice, we observed a reduction in the number of PV neurons when compared to Pv-Cre^+/−^ Shank3^Wt/Wt^ mice (Fig. 5B). However, in the visual cortex, no discernible difference was observed between Pv-Cre^+/−^ Shank3^Wt/Fl^ and Pv-Cre^+/−^ Shank3^Wt/Wt^ mice (Fig. 5C). Similar results were also obtained when we focused on the cells that express both TdTomato and Parvalbumin (Tdt + Pv + cells) (Additional file 1: Fig. 5) confirming the Cre specificity.

### Potentiating inhibitory activity reverses behavioral deficits caused by Shank3 deletion

**Fig. 6:**
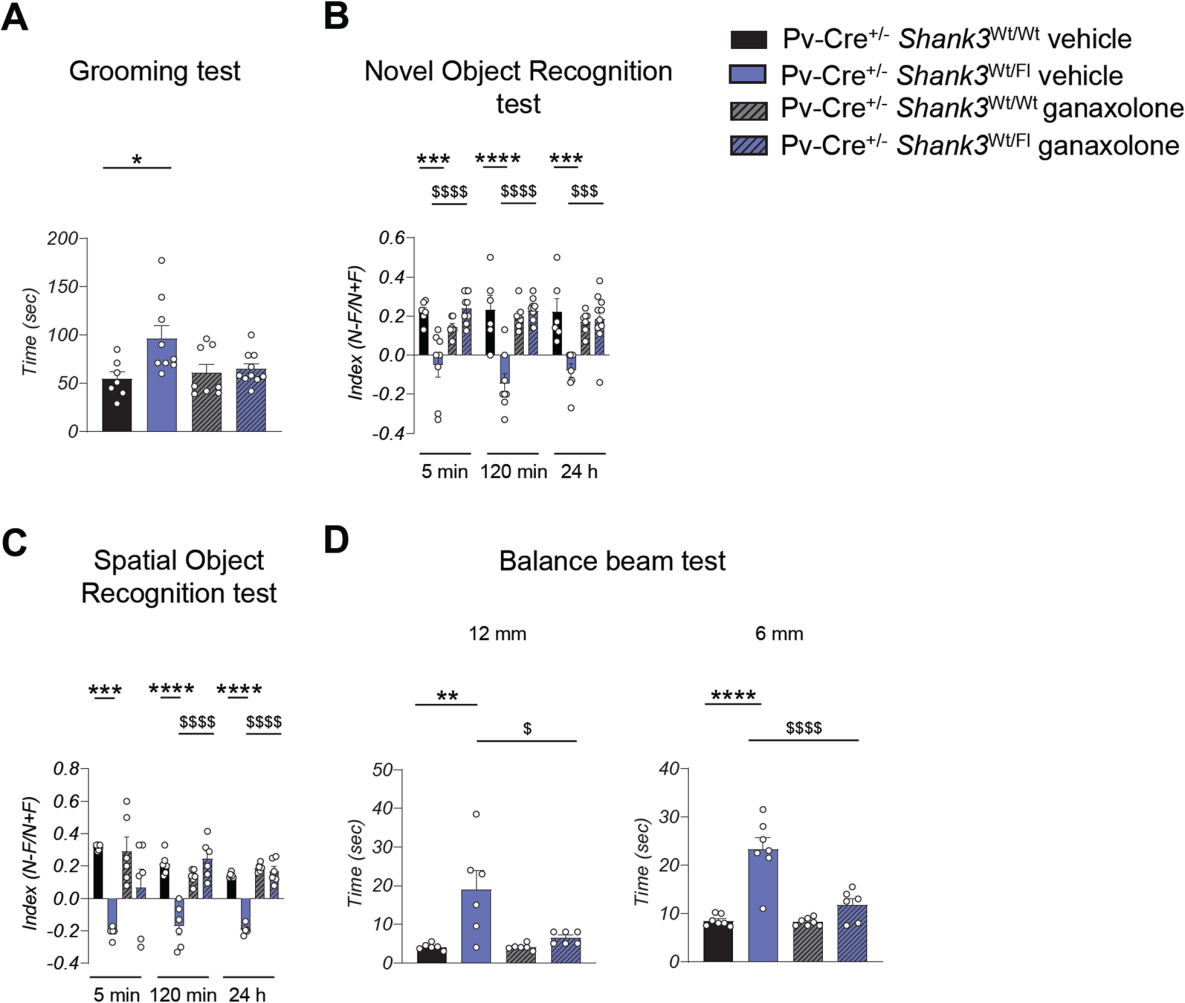
Ganaxolone pharmacological treatment rescues the behavioral alteration in Pv-Cre^+/−^ *Shank3* ^Fl/Wt^ mice. **A** Repetitive behavior was evaluated as the time spent doing grooming. Grooming was analyzed by two-way ANOVA; *n* = 7 Pv-Cre^+/−^ *Shank3*^Wt/Wt^ vehicle, *n* = 8 Pv-Cre^+/−^ *Shank3*^Wt/Wt^ ganaxolone, *n* = 9 Pv-Cre^+/−^ *Shank3*^Fl/Wt^ vehicle, *n* = 10 Pv-Cre^+/−^ *Shank3*^Fl/Wt^ ganaxolone; * p < 0.05 Pv-Cre^+/−^ *Shank3*^Wt/Wt^ vehicle compared with Pv-Cre^+/−^ *Shank3*^Fl/Wt^ vehicle. **B** Ganaxolone treatment rescues the novel object recognition memory of Pv-Cre^+/−^ *Shank3*^Fl/Wt^ mice. Novel object was analyzed by two-way ANOVA; *n* = 6 Pv-Cre^+/−^ *Shank3*^Wt/Wt^ vehicle, *n* = 7 Pv-Cre^+/−^ *Shank3*^Wt/Wt^ ganaxolone, *n* = 8 Pv-Cre^+/−^ *Shank3*^Fl/Wt^ vehicle, *n* = 10 Pv-Cre^+/−^ *Shank3*^Fl/Wt^ ganaxolone; *** p < 0.001, **** p < 0.0001 Pv-Cre^+/−^ *Shank3*^Wt/Wt^ vehicle compared with Pv-Cre^+/−^ *Shank3*^Fl/Wt^ vehicle; $$$* p* < 0.001; $$$$* p* < 0.0001 Pv-Cre^+/−^ *Shank3*^Fl/Wt^ vehicle compared with Pv-Cre^+/−^ *Shank3*^Fl/Wt^ ganaxolone. **C** Spatial memory was evaluated by determining a discrimination index in the spatial object recognition test. Data were analyzed by two-way ANOVA; *n* = 6 for each group; *** p < 0.001, **** p < 0.0001 Pv-Cre^+/−^ *Shank3*^Wt/Wt^ vehicle compared with Pv-Cre^+/−^ *Shank3*^Fl/Wt^ vehicle; $$$$* p* < 0.0001 Pv-Cre^+/−^ *Shank3*^Fl/Wt^ vehicle compared with Pv-Cre^+/−^ *Shank3*^Fl/Wt^ ganaxolone. **D** Motor coordination was evaluated as the time spent to cross the beam with a flat surface of 12 or 6 mm width. All p-values were derived using two-way ANOVA; *n* = 6 for each group in the 12 mm test; *n* = 7 Pv-Cre^+/−^ *Shank3*^Wt/Wt^ vehicle, *n* = 7 Pv-Cre^+/−^ *Shank3*^Wt/Wt^ ganaxolone, *n* = 7 Pv-Cre^+/−^ *Shank3*^Fl/Wt^ vehicle, *n* = 6 Pv-Cre^+/−^ *Shank3*^Fl/Wt^ ganaxolone in the 6 mm balance test; ** p < 0.01, **** p < 0.0001 Pv-Cre^+/−^ *Shank3*^Wt/Wt^ vehicle compared with Pv-Cre^+/−^ *Shank3*^Fl/Wt^ vehicle; $* p* < 0.05; $$$$* p* < 0.0001 Pv-Cre^+/−^ *Shank3*^Fl/Wt^ vehicle compared with Pv-Cre^+/−^ *Shank3*^Fl/Wt^ ganaxolone

We discovered that the deletion of Shank3 in PV neurons leads to brain hyperexcitability. To investigate whether positive allosteric modulation of GABA_A_ receptors by the neurosteroid ganaxolone could improve the deficits in Pv-Cre^+/−^ *Shank3*^Wt/Fl^ mice we administered ganaxolone (5 mg/Kg) or vehicle intraperitoneally to mice before conducting behavioral tests. Due to their broad availability and safety, benzodiazepines and other positive allosteric modulators of GABA_A_ receptors could be a near-term strategy to improve the symptoms of PMS patients. However, one difficulty in using benzodiazepines is their sedative properties. For this reason, for the in vivo experiments we chose ganaxolone because it has fewer side effects. We first assessed whether ganaxolone could ameliorate stereotyped behavior by performing the grooming test and found that ganaxolone administration resulted in a significant reduction in grooming time compared to the vehicle-injected mice (Fig. 6A). In addition, ganaxolone treatment improved memory deficits in Pv-Cre^+/−^ *Shank3*^Wt/Fl^ mice as shown by the results of novel object recognition test (Fig. 6B) and spatial object recognition test (Fig. 6C). Moreover, ganaxolone treatment rescued motor coordination deficits in Pv-Cre^+/−^ *Shank3*^Wt/Fl^ (Fig. 6D).

**Fig. 7:**
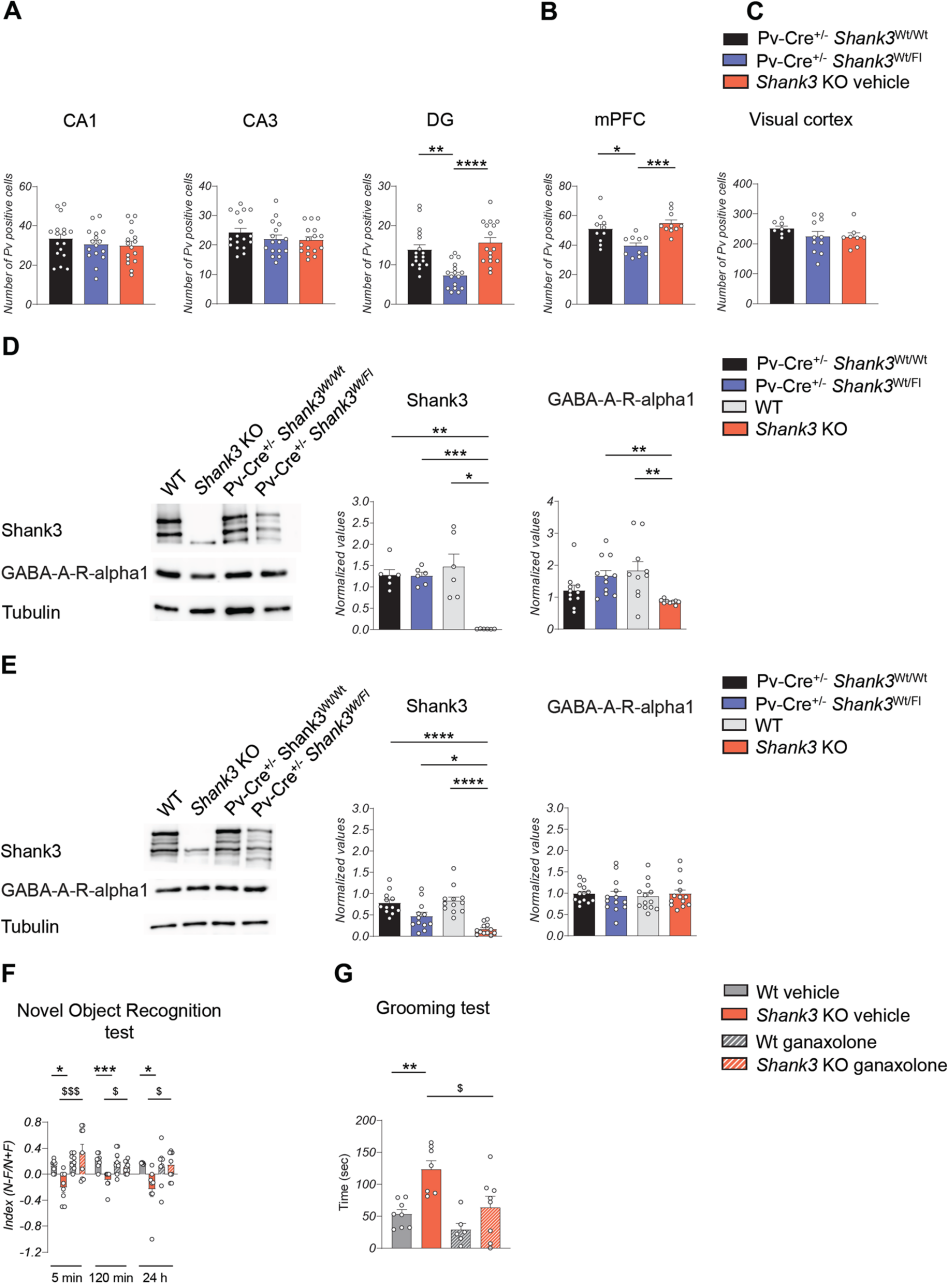
Effect of ganaxolone on *Shank3* KO mice. **A** Quantification of the number of PV neurons in the hippocampus. *Shank3* KO mice did not present alteration in the number of Parvalbumin interneurons. Data from the CA1 were analyzed by one-way ANOVA; quantification of the CA3 and DG was analyzed by Kruskal–Wallis test; *n* = 17 Pv-Cre^+/−^ *Shank3*^Wt/Wt^, *n* = 16 Pv-Cre^+/−^ *Shank3*^Fl/Wt^, *n* = 16 *Shank3 KO*; ** p < 0.01; *** p < 0.001; *n* = number of bilateral slides analyzed; 3 animals used for each group; CA1 = Cornu Ammonis-1; CA3 = Cornu Ammonis-3; DG = dentate gyrus. **B** Quantification of the number of PV neurons in the medial prefrontal cortex were analyzed by one-way ANOVA; *n* = 10 Pv-Cre^+/−^ *Shank3*^Wt/Wt^, *n* = 10 Pv-Cre^+/−^ *Shank3*^Fl/Wt^, *n* = 10 *Shank3* KO; *n* = number of bilateral slides analyzed; 3 animals used for each group. **C** Quantification of the number of PV neurons in the visual cortex were analyzed by one-way ANOVA; *n* = 8 Pv-Cre ± Shank3Wt/Wt, *n* = 11 Pv-Cre ± Shank3Fl/Wt, *n* = 8 Shank3 KO. *n* = number of bilateral slides analyzed. **D** Representative western blots and relative protein quantification from hippocampal PSD-enriched fraction derived from adult mice. Data obtained from the quantification of Shank3 were analyzed by Brown-Forsythe and Welch ANOVA; GABA-A-R-alpha1 was analyzed by Kruskal–Wallis test. Shank3 *n* = 6 for each group; GABA-A-R-alpha1 *n* = 11 Pv-Cre^+/−^ *Shank3*^Wt/Wt^ and Pv-Cre^+/−^ *Shank3*^Fl/Wt^, *n* = 10 *Shank3* WT, *n* = 9 *Shank3* KO. * p < 0.05; ** p < 0.01; *** p < 0.001. **E** Representative western blots and relative protein quantification from cortical PSD-enriched fraction derived from adult mice. Data obtained from the quantification of Shank3 was analyzed by Brown-Forsythe and Welch ANOVA. GABA-A-R-alpha1 protein quantification was analyzed by Kruskal–Wallis test. Shank3 *n* = 12 for each group; GABA-A-R-alpha1 *n* = 13 for each group. * p < 0.05; **** p < 0.0001. **F** Evaluation of evocative memory in Wt and *Shank3* KO mice after ganaxolone treatment was evaluated by calculating a discrimination index in the novel object recognition test. Data were analyzed by two-way ANOVA; *n* = 8 Wt vehicle, *n* = 8 Wt ganaxolone, *n* = 8 *Shank3* KO vehicle, *n* = 9 *Shank3* KO ganaxolone; * p < 0.5; *** p < 0.001 Wt vehicle compared with *Shank3* KO vehicle; $* p* < 0.05; $$$* p* < 0.001 *Shank3* KO vehicle compared with *Shank3* KO ganaxolone. **G** Repetitive grooming behavior was analyzed by two-way ANOVA; *n* = 8 Wt vehicle, *n* = 6 Wt ganaxolone, *n* = 7 *Shank3* KO vehicle, *n* = 8 *Shank3* KO ganaxolone; ** p < 0.01 *Shank3* Wt vehicle compared with *Shank3* KO vehicle; $* p* < 0.05 *Shank3* KO vehicle compared with *Shank3* KO ganaxolone

Finally, to better characterize how Shank3 deletion in the entire brain affect PV neurons we quantified the number of PV neurons in the hippocampus, medial prefrontal cortex (mPFC), and visual cortex of Shank3 knockout (KO) mice. Surprisingly, we did not observe any significant differences in the number of PV neurons in these brain regions compared to wild-type (Wt) mice (Fig. 7A–C). However, we did observe a significant reduction in the expression of the GABA-A-R-alpha1 subunit in the postsynaptic density (PSD)-enriched fraction from the hippocampus of Shank3 KO mice compared to Wt and Pv-Cre^+/−^ *Shank3*^Wt/Fl^ (Fig. 7D), suggesting that ganaxolone treatment might have a beneficial effect also in *Shank3* KO mice. In PSD-enriched fraction from the cortex we did not detect any alteration in GABA-A-R-alpha1 subunit (Fig. 7E); moreover, similar to the total cortex, we did not observe any significant differences in GABA-A-R-alpha1 expression between the groups analyzed in PSD-enriched fraction from the visual cortex (data not shown). To evaluate the effects of ganaxolone treatment on Wt and Shank3 KO mice, we injected the mice with ganaxolone (5 mg/Kg i.p.) or vehicle 30 min before conducting behavioral tests. Our results demonstrate that ganaxolone treatment rescued memory impairment in *Shank3* KO mice at 5-min, 120-min and 24-h delay, as evidenced by the results of novel object recognition test (Fig. 7F). Additionally, acute treatment with ganaxolone was also able to rescue repetitive behavior in *Shank3* KO mice to the levels of Wt mice (Fig. 7G). In conclusion, our findings demonstrated that PV neurons activity is sensitive to *Shank3* ablation and that restoring their function with ganaxolone may represent a possible pharmaceutical approach for ameliorating Shank3-related behavioral alterations.

## Discussion

This study provides significant evidence demonstrating that Shank3 plays a crucial role in modulating the activity of inhibitory circuit in vivo. Our data indicate that *Shank3* deletion leads to a severe impairment in the recruitment of PV interneurons and that the deletion of Shank3 specifically in PV-positive neurons is sufficient to recapitulate some of the behavioral deficits observed in individuals with PMS, such as repetitive behavior, memory impairments and motor problems. Additionally, local field potential recordings of Pv-Cre^+/−^ *Shank3*^Wt/Fl^ demonstrated that these behavioral deficits are associated to cortical hyperexcitability. Finally, we showed that enhancing the activity of GABA_A_ receptors with ganaxolone may be a viable strategy for ameliorating some of the behavioral symptoms resulting from *Shank3* deletion. Previous research has shown that alterations in the balance between excitation and inhibition (E-I) are present in various models of neurodevelopmental disorders and have been strongly linked to epilepsy [17, 49–52]. Notably, at least a third of individuals with PMS develop epilepsy [3] which is consistent with the impairment of the PV inhibitory feedback observed in our study.

Although GABAergic neurons represent a minority cell type, they control the activity level of principal neurons in the brain. The loss or dysfunction of interneurons has been implicated in numerous neuropsychiatric disorders [49, 53–55] and multiple studies support the hypothesis that specific symptoms of ASDs may be caused by an increase in the ratio of excitatory to inhibitory synaptic transmission [56, 57].

Numerous studies have provided evidence that Shank family proteins are extensively expressed in GABAergic neurons where they seem to be crucial for their function and development [10, 58]. Preferential deletion of *Shank3* from interneurons has been linked to hyperexcitability of pyramidal neurons and increased sensitivity to sensory stimuli [19]. Recently, the visual cortex has emerged as a valuable model for evaluating cortical processing in mouse models of neurodevelopmental disorders [40–43, 59, 60]. Altered visual cortical function has been observed in Rett syndrome patients and in mouse models carrying mutations in MeCP2 or FOXG1, thereby supporting the notion that VEPs can serve as a dependable biomarker for assessing the pathological state of the brain [61, 62]. Although we did not observe spontaneous epileptic seizures in *Shank3* KO mice, we found a higher degree of excitability in anesthetized mice both in resting state and in response to visual stimulation. In resting state, the increased excitability of the *Shank3* KO mice manifested as up states that were longer and had higher power in the 25–80 Hz range. During up states, cortical cell membrane potential is depolarized to subthreshold level and cells are more excitable [63] as clearly demonstrated by the transients observed in the spectrograms (Fig. 1A). The increased duration of up states is reflected by an increased power in the 10–100 Hz range (Fig. 1B). Our findings align with those of a mouse model of Fragile X Syndrome, which also displayed longer up states and hyperexcitability [64]. In addition to studying slow-wave activity, we have also investigated gain control, a fundamental property of neurons that allows to scale the response to excitatory inputs by means of feed-forward inhibition, thus reducing the incremental change of the response as the input strength increases [28]. Our results reveal that contrast gain control is impaired in the *Shank3* KO mice, and we hypothesized that this is due to a reduction in the recruitment of inhibitory interneurons by excitatory neurons. Indeed, the administration of midazolam, a positive modulator of GABA currents, reversed the gain control deficit observed in Shank3 KO mice. Previous research has demonstrated that contrast sensitivity gain control is regulated by the feedback of fast-spiking PV interneurons that are pivotal regulators of contrast transfer function in V1 [45]. Interestingly, the changes in spectral power observed in the *Shank3* KO and in the Pv-Cre^+/−^ *Shank3*^Wt/Fl^ are complementary (Fig. 4A). In the Pv-Cre^+/−^ *Shank3*^Wt/Fl^ the spectral power in the gamma band is similar to that of the control group, while there is a clear increase in power at low (< 5 Hz) and high frequencies (> 100 Hz). The increased power at low frequencies is likely due to the larger amplitude of up states that determine a larger component of the fundamental harmonic of slow-wave activity. Furthermore, EEG monitoring in both animal models and humans has shown that some epileptic conditions are associated with enhanced slow oscillations at frequencies < 0.5 Hz [65, 66]. On the opposite end of the Fourier spectra, increased power at frequencies > 100 Hz may indicate the presence of an epileptic focus. Indeed, intracranial recordings from the epileptic hippocampus (both in animal and human models) have reported fast ripples associated with increased epileptogenicity [67].

Different studies have shown that Shank proteins play a significant role in regulating synaptic transmission in parvalbumin (PV) neurons. Specifically, Shank1 has been shown to be highly expressed in PV neurons and is involved in the regulation of excitatory synaptic transmission in PV basket cells [68]. Additionally, deletion of Shank2 in PV neurons has been found to cause hyperactivity, increased self-grooming, and suppressed brain excitation [69]. Similarly, Shank3 has been found to be expressed in PV neurons and its knockout has been associated with reduced numbers of PV neurons in the striatum of mice [25].

To directly address the roles of Shank3 in regulating E/I balance and better understand the mechanisms responsible for the hyperexcitable state observed in the *Shank3* KO mouse, we used a PV-restricted conditional knockout approach. Our findings revealed that cell type-specific heterozygous deletion of *Shank3* in PV neurons was sufficient to induce behavioral alterations in contrast to what we found in *Shank3* full KO mice that did not exhibit cognitive defects in heterozygosis [30], strengthening the importance of the modulation of E-I balance during brain maturation [70]. Our study revealed that conditional deletion of *Shank3* in PV neurons caused an increase in repetitive behavior, reduced memory performance and alterations in motor coordination.

Surprisingly, the selective knockdown of *Shank3* in PV neurons did not significantly affect social behavior, despite the crucial role played by PV-positive neurons in regulating social behavior [68, 71, 72] and the strong social deficits observed in *Shank3* KO mice [30]. This suggests that the expression of *Shank3* in PV cells may not be critical for regulating social behavior in mice, or that compensatory mechanisms may be at play in the conditional knockout (cKO) mice, which could mask the effects of *Shank3* deletion in PV cells on social behavior. It is possible that other GABAergic or excitatory neurons could compensate for the loss of *Shank3* in PV cells, thereby maintaining social behavior in the cKO mice. Interestingly, a similar observation has been made regarding Shank2, where the deletion of *Shank2* specifically in PV-positive neurons has minimal impact on social interaction and communication, despite global *Shank2* knockout mice exhibiting pronounced impairments in social behavior [69]. This highlights the complexity of the role of Shank proteins in regulating behavior and the need for further investigation to fully understand their functions in different neuronal populations.

The electrophysiological phenotype of the Pv-Cre^+/−^ *Shank3*^Wt/Fl^ mice is complex. Up states have a larger amplitude with a high spectral power only at frequencies > 100 Hz compared to *Shank3* KO mice. However, Pv-Cre^+/−^ *Shank3*^Wt/Fl^ mice don’t exhibit increased power in the 25–80 Hz gamma band range compared to Wt (Fig. 4). We frequently observed large amplitude events (> 1 mV) suggestive of hypersynchronous, interictal events. Thus, the partial deletion of *Shank3* in a subset of cortical neurons causes a more severe hyperexcitable phenotype than the complete KO. On the other hand, the two models have subtle differences in phenotype: the functional and behavioral phenotype in the Pv-Cre^+/−^ *Shank3*^Wt/Fl^ mouse is associated with hyperexcitability, while the *Shank3* KO mouse presents a more complex ASD-like picture but no clear signs of hyperexcitability except for the deficit in gain control. A possible interpretation of this difference is that the loss of *Shank3* may lead to impairments in excitatory synapses, which in the complete KO mouse, could result in a reduction of excitatory drive on both pyramidal neurons and interneurons, thus partially compensating for the E-I balance. In contrast, the PV model with *Shank3* deletion only in PV interneurons has reduced excitatory drive on these specific interneurons, leading to a stronger imbalance toward excitation. However, further studies are needed to fully understand the mechanisms underlying the observed differences in phenotype between these two models.

The reduction in PV neurons observed in the hippocampus and mPFC of PV-Cre^+/−^ Shank3^Wt/Fl^ mice highlights the importance of GABAergic signaling in proper brain development and function. This finding also suggests that compounds targeting deficits in the GABAergic system, such as ganaxolone, may have therapeutic potential for treating brain disorders characterized by E/I imbalances, such as ASD [57, 73]. Indeed, the administration of midazolam showed that pharmacological reinforcement of inhibition was sufficient to restore gain control in *Shank3* KO mice, possibly normalizing the E/I imbalance. However, although being effective GABA_A_ receptor agonists, benzodiazepines often cause side effects including sedation. Ganaxolone is a synthetic neurosteroid that acts as a positive allosteric modulator of GABAA receptors, enhancing the activity of the inhibitory neurotransmitter GABA. Importantly, ganaxolone has been shown to be effective in reducing seizures and improving behavior in animal models of ASD [74, 75]. Additionally, ganaxolone has a more favorable side effect profile than benzodiazepines [76, 77] making it a promising candidate for clinical use. We demonstrated that a single dose of ganaxolone was effective in restoring normal behavior in Pv-Cre^+/−^ *Shank*3^Wt/Fl^ mice. Additionally, while we did not observe any changes in the number of PV neurons in *Shank3* KO mice, our results showed that *Shank3* knockdown led to a significant reduction in the expression of GABA-A-R-alpha1 in the hippocampus. Treatment with ganaxolone improved memory deficits and repetitive behaviors in *Shank3* KO mice, indicating that modulation of the GABAergic system may be a viable therapeutic strategy for individuals with Shank3 mutations.

Although a more comprehensive molecular characterization remains to be determined, our results suggest that that targeting the GABAergic system, specifically by enhancing GABAergic transmission through allosteric modulation of GABA_A_ receptors, may represent a potential therapeutic strategy for treating some symptoms associated with Shank3-related disorders. Our study demonstrated that Shank3 is involved in the modulation of PV neurons activity and emphasize the challenges and importance of defining the specific neuronal population and circuits causative of behavioral deficits due to *Shank3* deletion in order to develop effective therapies for PMS and ASD.

Future studies should focus on identifying the molecular mechanisms underlying the effects of *Shank3* deletion on PV neurons and its impact on other GABAergic neurons. Finally, chronic treatment with ganaxolone should be further evaluated to assess its long-term effects on behavior in *Shank3* KO mice.

## Limitations

A major limitation of this study is that the neurobiological mechanisms linking *Shank3* deletion in PV neurons and behavioral alterations remain to be investigated. While our study provides evidence for a specific *E*/*I* imbalance and cortical hyperexcitability, more comprehensive electrophysiological and molecular analyses of PV inhibitory neurons in different brain areas are necessary to establish a causal link between cellular and synaptic deficits and ASD-like behaviors. Additionally, although PV neurons make up 50% of GABAergic cells, the role of Shank3 in other classes of inhibitory neurons should be explored in future studies. Finally, while our study suggests that ganaxolone may represent a promising therapy for PMS and ASD, further studies are required to fully characterize its efficacy and safety profile. Additionally, future research is needed to investigate the effects of ganaxolone administration during brain development and in other *Shank3* KO mouse models and in the full Shank3 KO mouse model [78].

## Conclusions

Overall, our findings contribute to a better understanding of the role of Shank3 in the pathophysiology of PMS and ASD and pave the way for the development of new therapeutic strategies targeting the GABAergic system. However, further investigations are needed to fully elucidate the cellular and molecular mechanisms underlying Shank3-related synaptic deficits and to assess the efficacy of chronic treatment with ganaxolone or other GABAergic modulators in treating these disorders. Ultimately, these efforts may lead to the development of more effective and targeted treatments for PMS and ASD.

## Supplementary Information

**Additional file 1**. Supplementary materials. **Suppl. Figure 1:** Analysis of up states in Shank3 KO versus Wt mice. **Suppl. Figure 2:** Disrupted gain control in Shank3 KO mice: a further analysis. **Suppl. Figure 3:** Spectral analysis in Shank3 KO mice superfused with midazolam. **Suppl. Figure 4:** Shank3 full deletion specifically in inhibitory PV neurons does not exacerbate the phenotype. **Suppl. Figure 5:** Decrease of inhibitory interneurons in Pv-Cre+/- TdTomatoFl/- Shank3Fl/Wt mice.

## Data Availability

All data are available in the main text or the Additional files. The materials that support the findings of this study are available from the corresponding author upon reasonable request.

## Abbreviations

ASD: Autism spectrum disorder
KO: Knockout
LFP: Local field potential
PMS: Phelan–McDermid syndrome
PV: Parvalbumin-positive neurons
VEPs: Visual evoked potentials
Wt: Wild-type
