# Supplementary material for: Shank3 deletion in PV neurons is associated with abnormal behaviors and neuronal functions that are rescued by increasing GABAergic signaling
Source: Mol Autism. 2023 Aug 1;14:28. doi: 10.1186/s13229-023-00557-2 (PMC10394945; doi:10.1186/s13229-023-00557-2)
Supplement: Supplementary file 1 — Additional file 1. Supplementary materials. Suppl. Figure 1: Analysis of up states in Shank3 KO versus Wt mice. Suppl. Figure 2: Disrupted gain control in Shank3 KO mice: a further analysis. Suppl. Figure 3: Spectral analysis in Shank3 KO mice superfused with midazolam. Suppl. Figure 4: Shank3 full deletion specifically in inhibitory PV neurons does not exacerbate the phenotype. Suppl. Figure 5: Decrease of inhibitory interneurons in Pv-Cre+/- TdTomatoFl/- Shank3Fl/Wt mice. [file 13229_2023_557_MOESM1_ESM.pdf]

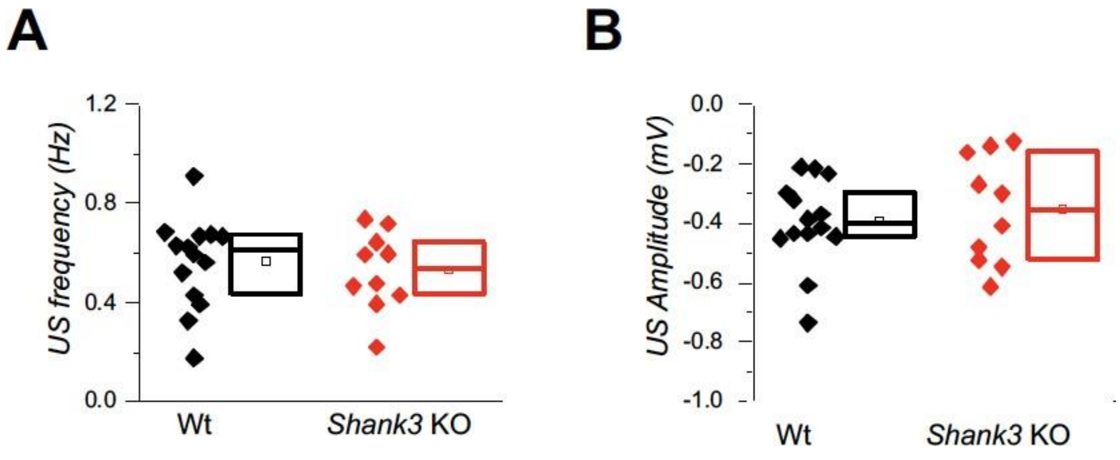

Suppl. Figure 1: Analysis of up states in *Shank3* KO versus Wt mice. Analysis of slow-wave activity reveals no differences in US frequency (A) and amplitude (B) (N=14 Wt, N=11 *Shank3* KO).

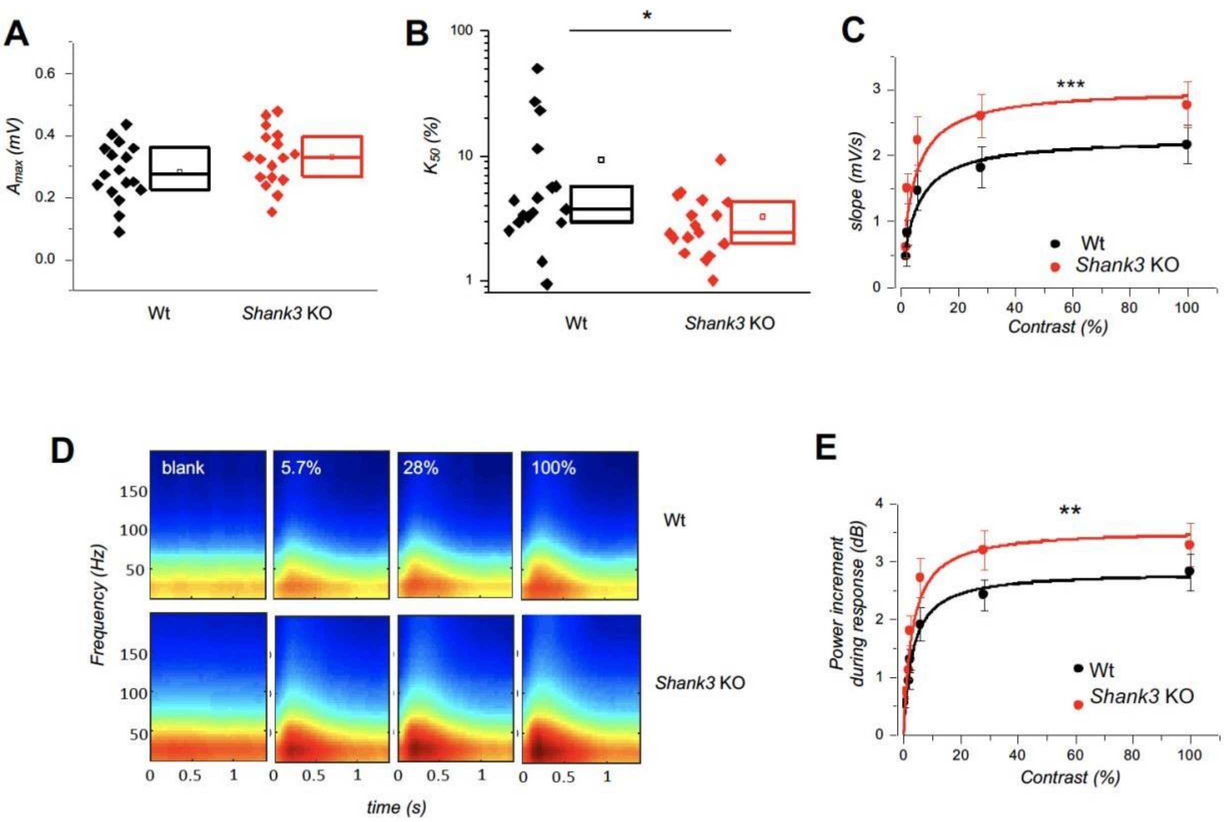

Suppl. Figure 2: Disrupted gain control in *Shank3* KO mice: a further analysis. (A-C) The contrast sensitivity curves have been fit with a Michaelis-Menten function (see Methods), with parameters A_{max} and k_{50}, which are here compared between *Shank3* KO and Wt mice. (A) While the maximum amplitude of response A_{max} did not show any difference (Mann-Whitney test), the half-saturating contrast k_{50} is significantly higher in *Shank3* KO mice (B; Mann-Whitney test; *$p<0.05$). In box plots in A and B, horizontal lines in the box represent the interquartile range and the median, while mean is the empty square. (C) Analysis of slope (explained in Methods) shows the same trend of contrast sensitivity in *Shank3* KO versus Wt mice indicating a more rapid response to visual stimulation in *Shank3* KO mice (Two-Way ANOVA, post-hoc Holm-Sidak test; ***$p<0.001$). (D) Short-time fast Fourier Transform for Wt (N=17) and *Shank3* KO mice (N=17) shows an enhanced power in response to visual stimuli. (E) Gamma band Power (25-80Hz) is increased at all contrasts during response (Two-Way ANOVA, post-hoc Holm-Sidak test; **P=0.001).

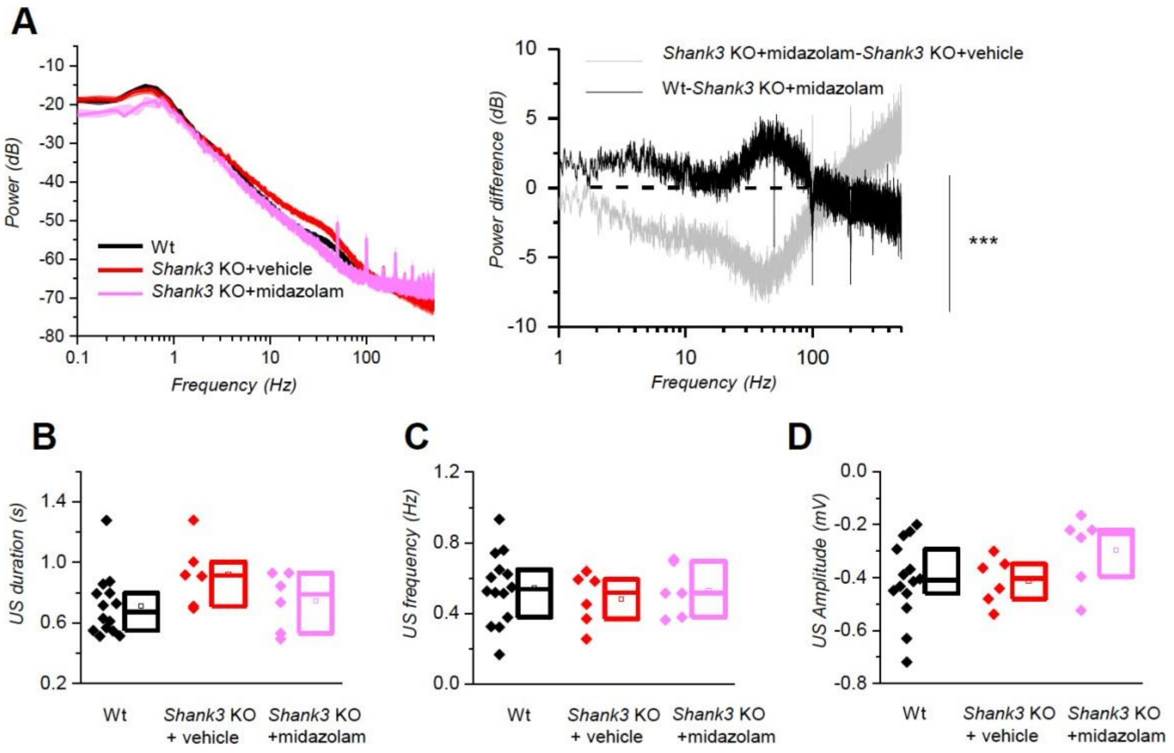

Suppl. Figure 3: Spectral analysis in *Shank3* KO mice superfused with midazolam. (A) (*left*) Spectral power in resting state show that the superfusion of midazolam on *Shank3* KO mice reduces the LFP power in the 5-80Hz band compared to vehicle (*right*). Power spectra difference is well evident in the grey curve in Shank3 KO mice treated with vehicle or after midazolam superfusion, while no difference is present in Wt mice compared to *Shank3* KO mice superfused with midazolam (Mann-Whitney rank test, *** p<0.001). (B,C,D) Analysis of slow-wave activity shows that there is no difference in US duration (B), frequency (C) and amplitude (D) between Wt, *Shank3* KO and *Shank3* KO mice superfused with midazolam.

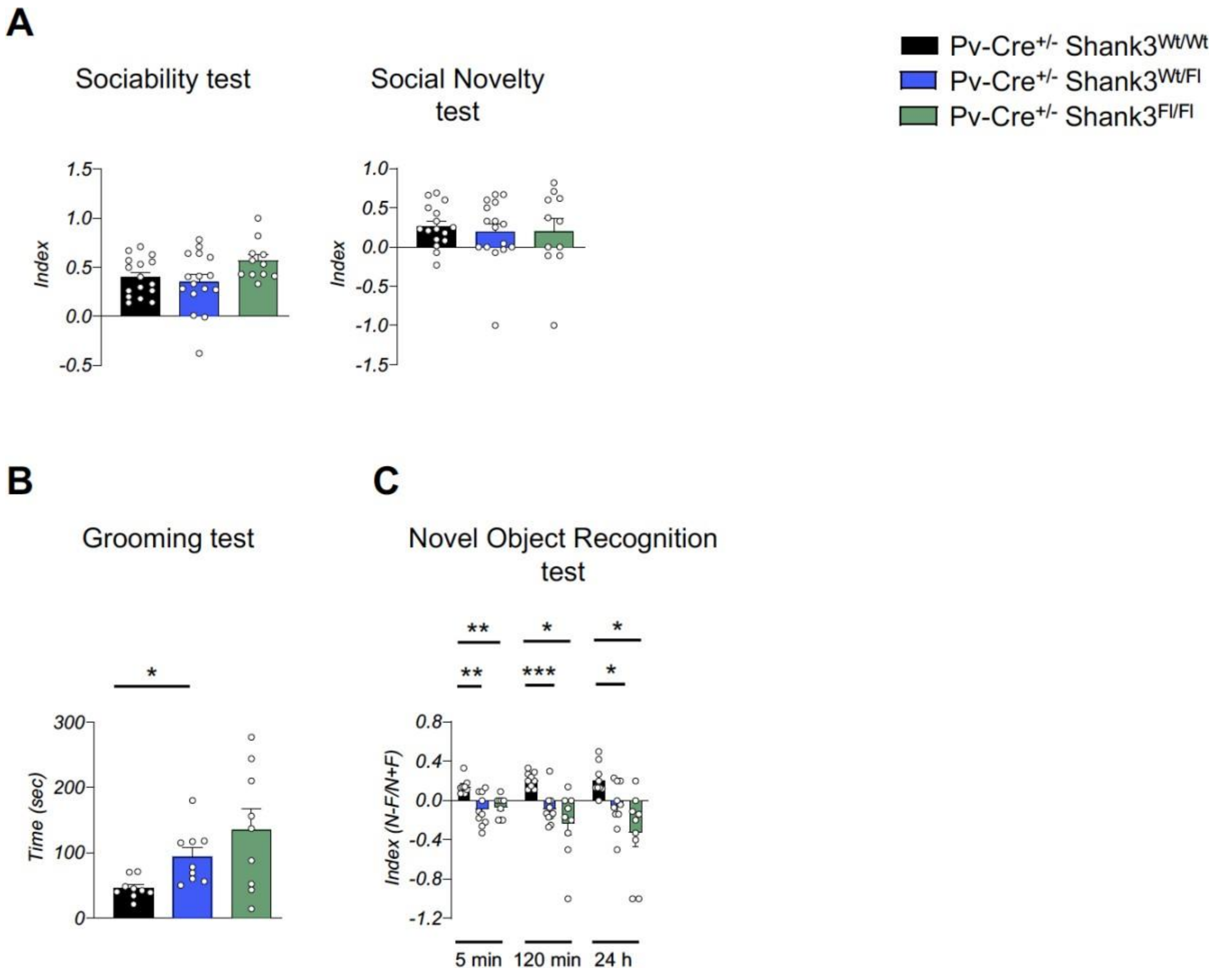

Suppl. Figure 4: *Shank3* full deletion specifically in inhibitory PV neurons does not exacerbate the phenotype. (A) Social interaction was evaluated by the three-chamber assays. Data from the sociability test were analyzed by One-way ANOVA; social novelty test were analyzed by Kruskal-Wallis test; n=16 Pv-Cre$^{+/-}$ *Shank3*$^{Wt/Wt}$, n=16 Pv-Cre$^{+/-}$ *Shank3*$^{Fl/Wt}$, n=11 Pv-Cre$^{+/-}$ *Shank3*$^{Fl/Fl}$. **(B)** Time spent doing grooming was analyzed by Brown-Forsythe and Welch ANOVA test; n=9 for each group; *p<0.05. (**C**) Memory impairment was evaluated with the novel object recognition test. Novel object recognition at 5 minutes was analyzed by Kruskal-Wallis test; novel object recognition at 120 minutes and 24 hours were analyzed by Brown-Forsythe and Welch ANOVA test; n=9 Pv-Cre$^{+/-}$ *Shank3*$^{Wt/Wt}$, n=10 Pv-Cre$^{+/-}$ *Shank3*$^{Fl/Wt}$, n=9 Pv-Cre$^{+/-}$ *Shank3*$^{Fl/Fl}$; *p<0.05; **p<0.01; ***p<0.001.

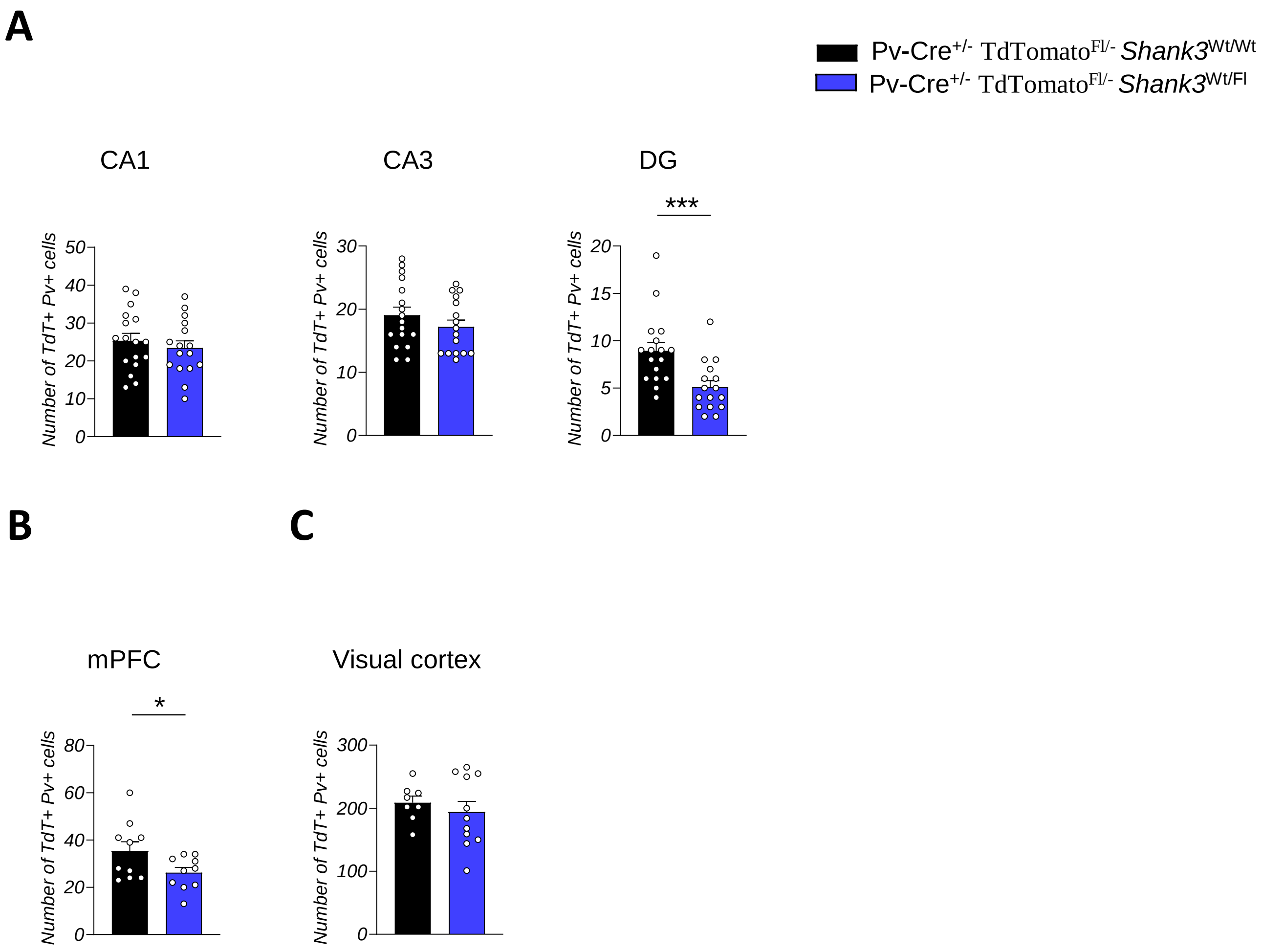

Suppl. Figure 5: Decrease of inhibitory interneurons in Pv-Cre$^{+/-}$ TdTomato$^{Fl/-}$ *Shank3*$^{Fl/Wt}$ mice. (A) Quantification of the number of cells positive for both TdTomato and Parvalbumin immunostaining in the hippocampus. Pv-Cre$^{+/-}$ TdTomato$^{Fl/-}$ *Shank3*$^{Fl/Wt}$ mice show a reduction of Parvalbumin interneurons in the dentate gyrus of the hippocampus. Data from the CA1 were analyzed by unpaired, two-tailed student's t-test; quantification of the CA3 and DG were analyzed by two-tailed Mann-Whitney test; n=17 Pv-Cre$^{+/-}$ TdTomato$^{Fl/-}$ *Shank3*$^{Wt/Wt}$, n=16 Pv-Cre$^{+/-}$ TdTomato$^{Fl/-}$ *Shank3*$^{Fl/Wt}$; ***p<0.001; n= number of bilateral slides analyzed; 3 animals used for each group. CA1= Cornu Ammonis-1; CA3= Cornu Ammonis-3; DG= dentate gyrus. **(B)** Quantification of the number of cells positive for both TdTomato and Parvalbumin immunostaining in the medial prefrontal cortex (mPFC). Data were analyzed by unpaired, two-tailed student's t-test; n=10 for each group; *p<0.05; n= number of bilateral slides analyzed; 3 animals used for each group. **(C)** Quantification of the number of cells positive for both TdTomato and Parvalbumin immunostaining in the visual cortex. Data were analyzed by unpaired, two-tailed student's t-test; n=8 Pv-Cre$^{+/-}$ TdTomato$^{Fl/-}$ *Shank3*$^{Wt/Wt}$, n=11 Pv-Cre$^{+/-}$ TdTomato$^{Fl/-}$ *Shank3*$^{Fl/Wt}$; n= number of bilateral slides analyzed; 3 animals used for each group.
